# Global coastal wave storminess

**DOI:** 10.1038/s41598-024-51420-0

**Published:** 2024-02-14

**Authors:** Hector Lobeto, Alvaro Semedo, Gil Lemos, Ali Dastgheib, Melisa Menendez, Roshanka Ranasinghe, Jean-Raymond Bidlot

**Affiliations:** 1 IHCantabria - Instituto de Hidráulica Ambiental de la Universidad de Cantabria, Santander, Spain; 2 Department of Coastal and Urban Risk and Resilience, IHE Delft Institute for Water Education, Delft, The Netherlands; 3 Instituto Dom Luiz (IDL), Faculdade de Ciências, Universidade de Lisboa, Lisbon, Portugal; 4 IMDC (International Marine and Dredging Company), Antwerp, Belgium; 5 Department of Infrastructure Engineering, University of Melbourne, Melbourne, Australia; 6 Department of Resilient Ports and Coasts, Deltares, Delft, The Netherlands; 7 European Centre for Medium-Range Weather Forecasts, Reading, UK

**Keywords:** Natural hazards, Physical oceanography

## Abstract

Coastal wave storms pose a massive threat to over 10% of the world’s population now inhabiting the low elevation coastal zone and to the trillions of $ worth of coastal zone infrastructure and developments therein. Using a ~ 40-year wave hindcast, we here present a world-first assessment of wind-wave storminess along the global coastline. Coastal regions are ranked in terms of the main storm characteristics, showing Northwestern Europe and Southwestern South America to suffer, on average, the most intense storms and the Yellow Sea coast and the South-African and Namibian coasts to be impacted by the most frequent storms. These characteristics are then combined to derive a holistic classification of the global coastlines in terms of their wave environment, showing, for example, that the open coasts of northwestern Europe are impacted by more than 10 storms per year with mean significant wave heights over 6 m. Finally, a novel metric to classify the degree of coastal wave storminess is presented, showing a general latitudinal storminess gradient. Iceland, Ireland, Scotland, Chile and Australia show the highest degree of storminess, whereas Indonesia, Papua-New Guinea, Malaysia, Cambodia and Myanmar show the lowest.

## Introduction

Coastal storms represent the ocean at its most energetic and violent state, with a considerable destructive power, impacting coastal areas and coastlines^1^. Coastal storms are related to strong winds, extreme wave heights, extreme sea levels, often together with intense precipitation, leading to coastal flooding and consequent damage, where not protected. Coastal storms are often the cause of substantial human^2,3^ and economic^4–7^ losses, as well as ecological disturbance and damage^8–10^. Nevertheless, coastal storms are part of the global weather pattern, and essential to the equilibrium of the climate system, bringing fresh water and nutrients to coastal ecosystems, contributing to their rejuvenation, and recharging coastal river catchments, underground aquifers and water reservoirs^1^.

This study focuses on a specific aspect of coastal storms: wind waves, henceforth referred to as coastal wave storms, or simply wave storms (or even just storms). Waves are usually separated into two categories: wind–sea and swell waves^11,12^. Wind–seas are waves in their generation process, under the effect of the undelaying winds. Swells are waves that have outrun the overlaying wind, propagating away from their generation area across long distances with minor attenuation^13,14^.

Coastal wave storms are powerful events in which the energy carried by the waves clearly exceeds the climatological mean values. Therefore, wave storms can have a significant impact on coastlines. Wave set-up, combined with storm surges and astronomical tides, can significantly increase the total water level at the coast^15^, inducing considerable and destructive coastal flooding^16–18^ or overtopping events^19^. In this regard, the wave contribution to coastal flooding can either be caused by wind–sea waves generated under low-pressure systems passing close to the coast such as during hurricane Katrina in 2005^20,21^, or by swell waves generated by remote storms, as in the wave storm event that impacted the tropical western Pacific islands^7,22^. Additionally, wave storms are considered a major coastal erosion driver^23,24^. It is worth mentioning that wave heights are not the only factor determining the impact of waves on the coast during wave storms. The wave set-up, for example, is dependent on both the wave period than on wave heights^25^. Also, the wave energy flux, determining the ability of waves to actually perform work^12^ on the coast (often called wave power), is a function of both the wave height and period, to the second and first orders, respectively^26^. Additionally, deviations of the predominant incoming wave direction during wave storm events, play a role in coastal erosion^27,28^.

Traditionally, wave storm investigations are based on significant wave height ($${H}_{s})$$ time series. The standard approach is based on identifying exceedances over a certain $${H}_{s}$$ threshold, where individual exceeding events are classified as wave storms. However, there is no standard or unique criterion in the literature to define this threshold. Its dependence on the local wave climate conditions, the availability of data and, to some extent, the intrinsic subjectivity of the author’s understanding of a wave storm, ends up determining the choice. The storm threshold has been defined according to different criteria such as the impact on the coast^29–31^, the agreement to the Generalized Pareto distribution^32–35^ or, more recently, through more complex statistical methods accounting for the joint distribution of the variables involved in the storm definition^36^. A pragmatic option for larger scale assessments is defining the threshold based on $${H}_{s}$$ statistics. It should also be noted that the geneses of the wave storms arriving at the coast are significantly different across different ocean basins, and from one ocean region to another^12,37–39^. The use of the $${H}_{s}$$ 95^th^ percentile^40–46^ or the combination of mean $${H}_{s}$$ and two $${H}_{s}$$ standard deviations^47–50^ are examples of different approaches that adapt well to the local climate.

Multiple studies have addressed the classification of the coastlines in terms of the characteristics of the storms recaching it. The energy of the storms has been considered as a pivotal element to conduct these classifications, as it is a key factor in the severity of the impact the storms may have at the coast. Studies differ on the metrics used to quantify the energy of the storms, from more simplistic ones, such as the storm power proposed by Dolan and Davis (1992)^51^ and used in multiple studies after^48,52^, to other metrics that consider the actual evolution over time of the energy impacting the coast^49,53–55^.

Nevertheless, all studies mentioned above have assessed wave storm conditions at local or, at the most, regional scales. To the extent of our knowledge, a thorough characterization and classification of coastal wave storms at a global scale has not been undertaken to date.

In the field of coastal science/engineering, knowledge of the dominant wave environment at a certain stretch of coast is important. Historically, what has been used in this regard are coarse wave environment classifications produced in the 1980s^56^ which essentially divides the world's coast into swell waves, storm waves and monsoon dominated areas. In the analysis of wave storms, it is important for the coastal science/engineering community to know whether, at a given location, storms are spread out through the year, occur in one concentrated season, or in a few seasons, the storm wave period and direction (and their variation within a year/among storm seasons), etc.

In this context, this study aims to provide an updated characterization of the wave storminess along the world’s coasts. Here, under the definition of wave storms as episodic wave events capable of altering the mean wave climatological conditions of a coastal stretch, the analysis is not restricted to those regions affected by *storm wave environments*^56^ but extended to all global coastlines. Thus, a global wave storminess classification based on a ~ 40-year robust, validated, global wave hindcast is produced, which provides a much more granular description of coastal wave storminess characteristics at global scale.

This study provides a quantitative and qualitative assessment of the global coastal wave storminess in which the main characteristics of wave storms are explored. New metrics to evaluate the wave storminess are proposed here for the first time, such as the relative importance of wind–sea storms versus swell storms, and the count of storm seasons at each coastal location. All this information is combined to provide a new holistic classification of the global coastlines in terms of the characteristics of wave storms, facilitating comparisons between different coastal regions. Moreover, a novel metric to qualitatively classify the degree of coastal wave storminess at a given location is presented, providing clear insights into regions that are more prone to be impacted by more potentially damaging storms.

## Results

### Assessment of coastal wave storm characteristics

The criterion used here to select coastal wave storms is based on the exceedances over a threshold of the ${H}_{s}$ time series (see “Methods”). To that end, a high-resolution global wave hindcast, produced with a recent European Centre for Medium-range Weather Forecasts (ECMWF) wave model version (ecWAM)^57^, forced with ERA-5 reanalysis winds and sea ice cover, is used (see “Methods”). For the sake of consistency and to allow direct comparisons between coastal regions, a single global threshold, the 95^th^ percentile *H*_*s*_, has been considered. Additionally, a sub-sample of wave storms, henceforth designated as severe wave storms, representing the most energetic wave storms, is separately analyzed in the study (see “Methods”).

Despite assessing the main characteristics of coastal wave storms globally is the main goal of this study, a brief description of the annual mean ${H}_{s}$ wave climate (1979 to 2020) along the global coastlines is also included in Supplementary Material to be used as a basis for further analyses and classification of the coastal wave storminess.

#### Frequency of occurrence and storm duration

**Figure 1:**
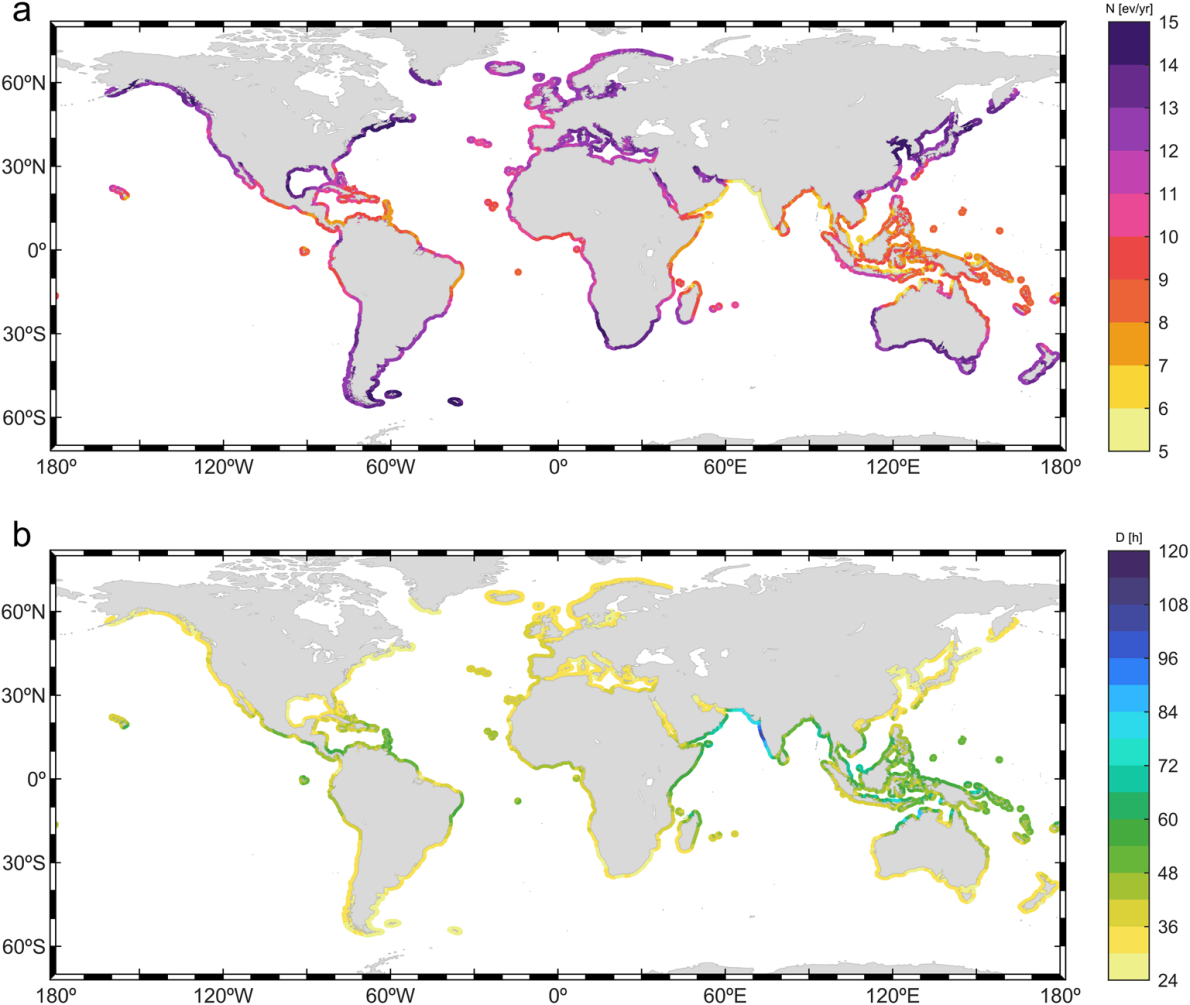
(**a**) Global coastal annual mean number of wave storms (in events/year—ev/yr) and (**b**) global coastal mean duration of wave storms (in hours) and (**b**) global coastal mean duration. The plots were generated using MATLAB R2023b (https://matlab.mathworks.com).

The frequency of occurrence relates to the number of impacting events. Thus, a higher number of events is likely to imply a higher number of coastal impacts. Figure 1 depicts the annual mean number of events, along with the mean duration of wave storms. The annual mean number of wave storms (Eq. 2) show significant heterogeneity along the global coastlines (Fig. 1a; results for severe storms are shown in Fig. SM10). Table 1 summarizes coastal regions showing the highest and lowest frequencies of occurrence of wave storms. Differences of more than 10 storm events per year can be observed among coastlines of different regions. Extratropical coastlines show a higher annual number of events compared to lower latitudes. The number of storm events poleward of 40° roughly exceeds 11 events per year in both hemispheres (Fig. 1a). Semi-enclosed seas (e.g., Gulf of Mexico, Mediterranean Sea, Sea of Japan), and marginal seas (e.g., Yellow Sea), also show high storm frequencies of more than 10 events per year. The mean annual number of storm events decreases along open coastlines closer to the equator, ranging between 5 and 11. Among them, the highest annual number of storm events is found along coastlines exposed to swells generated in the extratropical regions (e.g., the coasts of South Sumatra and Java). The lowest annual number of storm events are found in the coasts of the Arabian Sea.

**Table 1:** First column: Ranking order. Second and Fourth columns: coastal regions showing the top 10 highest and lowest frequencies of occurrence of wave storms along the global coastlines, respectively. Third and fifth columns: frequency of occurrence (mean number of events per year—ev/yr) of wave storms. (+) indicates that values could exceed the upper limit of the range.

Rank	Coastal region (highest)	*N* (ev/yr)	Coastal region (lowest)	*N* (ev/yr)
1	Yellow Sea coast	14–15 +	Arabian Sea coast	4–7
2	South-Africa & Namibia	13–15 +	North Java	5–8
3	Northeastern USA & Eastern Canada	12–15 +	Northwestern Australia	5–10
4	Gulf of Mexico coast	12–15 +	Eastern Malaysia	6–8
5	Southern—Western Australia	12–15 +	Northern Panama	6–8
6	Northeastern Japan	12–15 +	Eastern Somalia	6–9
7	Southeastern & southwestern South America	12–15 +	North Papua-New Guinea	6–9
8	Mediterranean Europe	11–14	Myanmar	6–9
9	Northwestern USA & Western Canada	11–14	Eastern Brazil	7–9
10	New Zealand	10–14	Lesser Antilles	7–9

**Figure 2:**
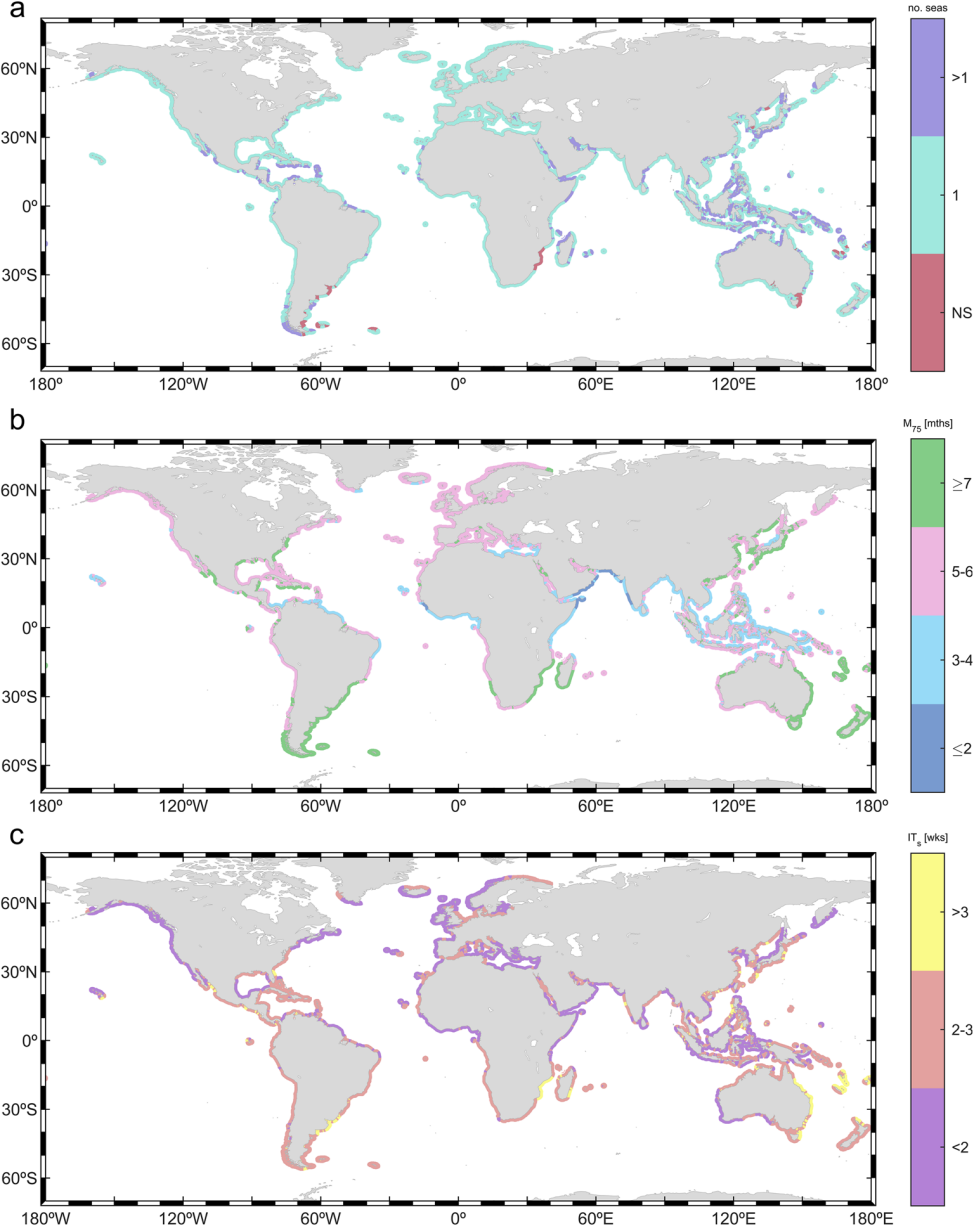
Global clustering of coastal wave storm events: (**a**) number of storm seasons (NS means no storm season), (**b**) number of months where at least 75% of wave storms occur (in months—mths), and (**c**) mean time between wave storms during the storm seasons (in weeks—wks). The plots were generated using MATLAB R2023b (https://matlab.mathworks.com).

**Figure 3:**
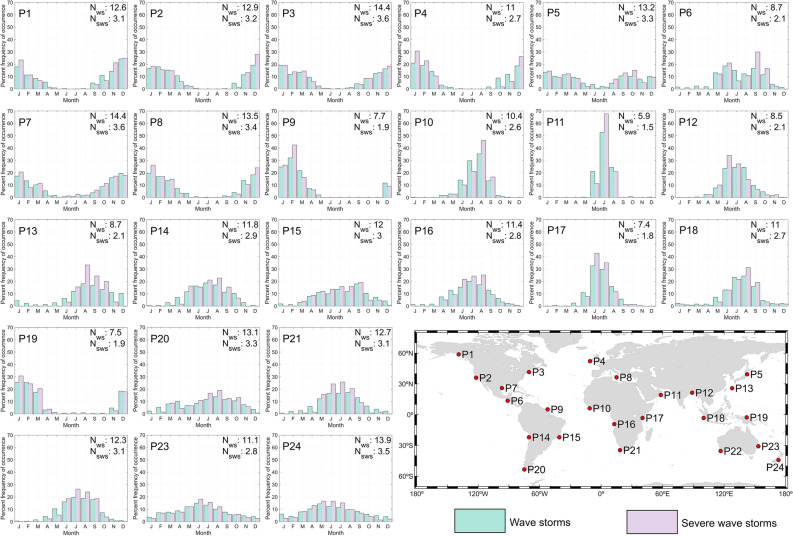
Monthly percentage frequency of occurrence of wave storm events at twenty-four key locations (P1 to P24: see map). Bars represent the monthly percentage frequency of occurrence of wave storms (green) and severe wave storms (purple). The plots were generated using MATLAB R2023b (https://matlab.mathworks.com).

The occurrence variability of wave storms within the year is addressed by assessing the mean yearly clustering of storm events (Fig. 2) and, for several key points (24), the variability of the monthly frequency of occurrence throughout the year (Fig. 3). Most coastlines (almost four-fifths) experience only one storm season (see “Methods”)—in other words, wave storms mostly occur in a unique continuous period of consecutive months. Other coastal locations, mostly in intertropical and subtropical latitudes, show more than one storm season (e.g., Eastern Japan, Northern Australia). Additionally, there are coasts where despite some (weak) seasonality, the number of storms remains unchanged all year round, i.e., there are no storm seasons (e.g., coast of Mozambique, east coast of Tasmania, southeastern coast of South America).

However, it is not only the number of events impacting the coasts that is relevant, but also the rate with which they reach the coast. More frequent storms can lead to more damage to the coast. If the calm period between storms shortens, beaches have less time to naturally recover before the arrival of the next event^58,59^. This may result in cumulative erosion^60,61^ and, consequently, decreased protection against flood events^62,63^.

Figure 2b shows the minimum number of months where at least 75% of the storms occur (i.e., M_75_; see “Methods”). Storms in the Northern Hemisphere (NH) extratropical coasts are, in general, concentrated in a shorter period (~ 4–6 months) compared to the Southern Hemisphere (SH; ≥ 6 months). For example, at coastal locations in the extratropical NH (e.g., P1-P4 in Fig. 3) storms mostly concentrate in the winter months, whereas at coastal locations in the extratropical SH (e.g., points P20–P24 in Fig. 3) a more even distribution across the year can be seen. This stronger NH extratropical seasonal pattern is clear when analyzing the seasonal frequency of occurrence of the events (Figs. SM11–SM12), showing that while in the NH most events occur during the boreal winter in DJF and almost none in JJA, SH coasts can still experience 1 or 2 storms in DJF. Tropical coasts show great heterogeneity in *M*_*75*_. Among them, the coasts of the Arabian Sea are those where wave storms are concentrated in a shorter period: only three months (key point P11), probably as a consequence of the summer Monsoon wind features^64^.

The average time between storms during the storm seasons (${IT}_{s}$, Eq. 4; note: along coasts with no storm seasons, this metric is computed considering the whole year) varies from less than 2 weeks to more than three weeks (Fig. 2c). In general, low inter-storm time periods are found along the northwestern coastlines of the continents (e.g., northwestern European coast). Note that this issue is especially relevant as these coasts also experience energetic wave climates (Fig. SM9).

The time for which storm conditions persist relates to the potential damage caused by these events^65^. The mean duration of wave storms and severe wave storms (D, Eq. 5) is shown in Fig. 1b (5–95th duration percentiles in Fig. SM13) and Fig. SM14, respectively. Additionally, the coastal regions showing the longest and the shortest durations are summarized in Table SM1. The spatial pattern of the mean storm duration is, in general terms, opposite to the mean number of events per year. The mean duration of wave storms impacting coasts in the extratropical regions of both hemispheres ranges between less than 24 h to more than 36 h (Fig. 1b). The extratropical region of both hemispheres shows similar histograms of storm durations (Fig. SM15). Thus, the most probable storm duration ranges between 12 and 24 h for extratropical key points, with the probability smoothly decreasing for longer durations (e.g., points P1–P5 for the NH and P20–P24 for the SH in Fig. SM15).

In the intertropical and subtropical latitudes, the mean wave storm duration is longer (average of 45 h between 35°S and 35°N). There are considerable differences between coasts, with minimum mean durations of around 36 h in, for example, the innermost coasts of the Gulf of Mexico and maximum values exceeding 120 h in eastern parts of the Arabian Sea coast, the latter being a consequence of the steadiness and duration of the Monsoon winds^64^. Accordingly, histograms in tropical key points differ significantly depending on the origin of the wave storms. Tropical coasts mainly affected by extratropical swell storms (e.g., P14, P16 in Fig. SM15) show a similar histogram to points located in the extratropical region (e.g., P21, P22 in Fig. SM15). On the other hand, tropical coasts affected at the same time by wave storms with different geneses: tropical cyclones (TCs), extratropical cyclones (ETCs) and trade winds, show more complex histograms characterized by multiple local maxima (e.g., P9, P19 in Fig. SM15).

The most energetic storms generated in the extratropical regions can last, on average, more than 48 h over the analyzed threshold. These extreme events, with enhanced destructive power, are notably long (> 60 h) along the west coast of Europe and USA. The most probable mean duration of severe storms in the extratropical region is higher than for regular wave storms, lying between 36 and 60 h. Consistently, the mean duration in the intertropical and subtropical regions also increases, showing minimum durations of 60 h along open coastlines and, particularly, longer than 132 h in most of the Arabian Sea coast.

#### Integrated wave climate parameters

The wave height, period and direction of wave storms play a key role in coastal processes such as flooding and erosion. Therefore, accurately assessing these values is crucial for determining potential impacts caused by storm events. The significant wave height of the storms is a primary driver of coastal erosion. Moreover, the incident angle of the waves and the period also influences sediment movement^66^. Regarding coastal flooding, both the wave period and the wave height play a prominent role in the set up generated by waves^25^, and hence, in the contribution of waves to extreme coastal total water levels. Also, the design of coastal structures is conditioned by the height, period and direction of incident wave storms^67–69^. This subsection examines the mean value of these parameters within storm events along the global coastlines.

**Figure 4:**
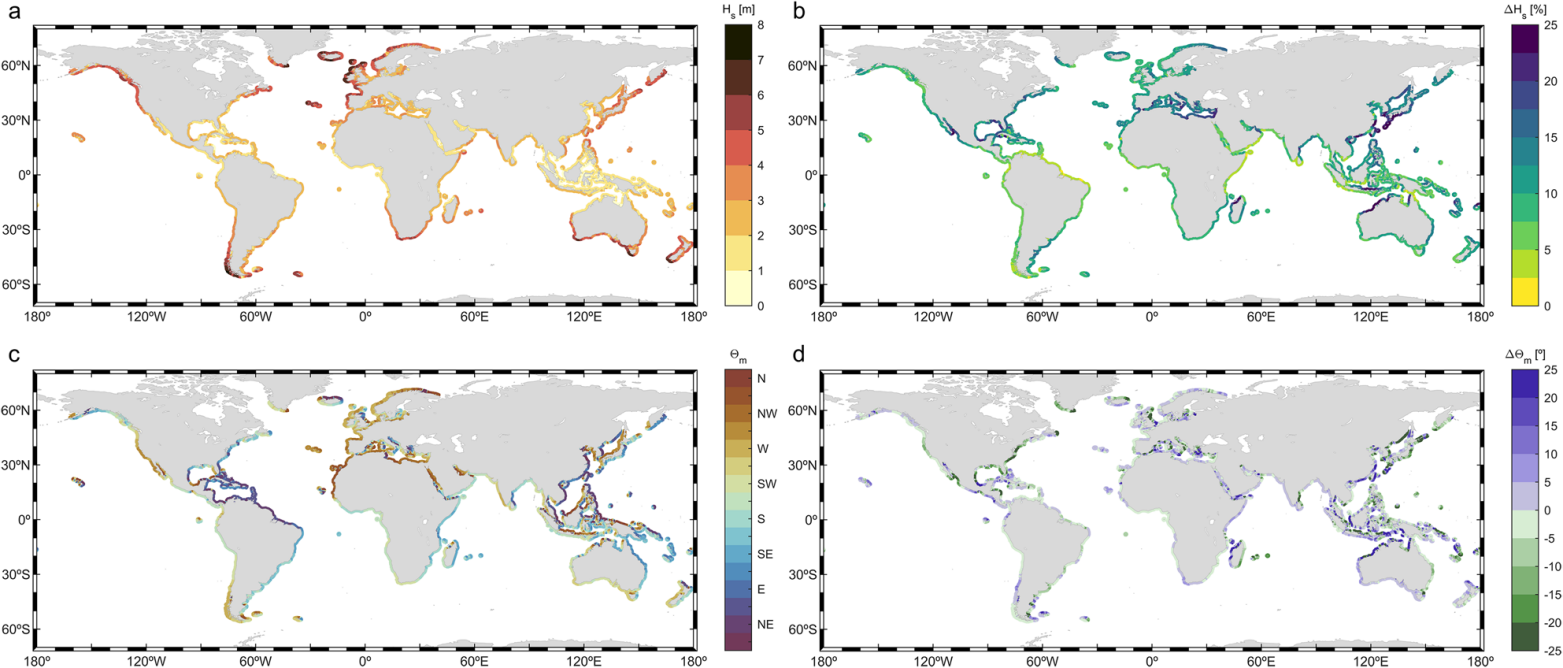
Global coastal (**a**) mean ${H}_{s}$ (in m) and (**c**) mean ${\theta }_{m}$ (in º) of wave storms. Global coastal increase in (**b**) mean ${H}_{s}$ (in %) and (**d**) mean ${\theta }_{m}$ (in º) for severe wave storms with respect to wave storms. The plots were generated using MATLAB R2023b (https://matlab.mathworks.com).

Figure 4 shows the mean values of ${H}_{s}$ and ${\theta }_{m}$ integrated wave parameters during wave storms and their change during severe wave storms. The mean ${H}_{s}$ of wave storms (Eq. 6) is shown in Fig. 4a (5–95th ${H}_{s}$ percentiles in Fig. SM21a-b). Additionally, Table 2 summarizes the coastal regions impacted by the highest and lowest wave storm events. The mean ${H}_{s}$ of wave storms shows a latitudinal gradient with higher values at extratropical latitudes, along the western coastlines of the continents. Severe wave storms show, in general, a similar mean ${H}_{s}$ pattern but, as expected, with higher magnitudes. In this regard, Fig. 4b depicts the increase in mean ${H}_{s}$ in severe storms compared to regular wave storms (the mean ${H}_{s}$ values for severe storms are shown in Fig. SM16), showing a heterogenous pattern along the global coastlines with relative increases ranging between less than 2% to more than 25%.

**Table 2:** First column: Ranking order. Second and fourth columns: top 10 coastal regions showing the highest and lowest mean ${H}_{s}$ along the global coastlines, respectively. Third and fifth columns: mean ${H}_{s}$ (meters—m) of wave storms and, between brackets, of severe wave storms. (+) indicates that values could exceed the upper limit of the range.

Rank	Coastal region (Highest)	${\mathbf{H}}_{\mathbf{s}}$(m)	Coastal region (lowest)	${\mathbf{H}}_{\mathbf{s}}$(m)
1	Northwestern Europe	4–8 + _(5–8+)_	Indonesian interior seas coasts	0–2_(0–3)_
2	Southwestern South America	4–8 + _(5–8+)_	Red Sea coast	0–2_(0–3)_
3	Southern Australia	3–7_(3–7)_	Northern Australia	0–3_(0–3)_
4	New Zealand	3–7_(3–7)_	Gulf of California coast	1–2_(1–3)_
5	Gulf of Alaska coast	3–6_(4–7)_	Gulf of Guinea coast	1–2_(1–3)_
6	Southwestern Europe	3–6_(4–7)_	Persian Gulf coast	1–2_(1–3)_
7	South Africa	3–6_(4–6)_	Northern Papua-New Guinea	1–2_(1–3)_
8	Eastern Canada	3–6_(3–6)_	Pacific Central America	1–2_(1–3)_
9	Northeastern Japan	3–6_(3–7)_	Yellow Sea coast	1–2_(1–4)_
10	NH Tropical western Pacific coast	2–5_(2–7)_	Gulf of Bengal coast	1–3_(1–4)_

The northwestern European coasts are impacted by wave storms characterized by high mean ${H}_{s}$ (e.g., the west coasts of Ireland and Scotland), and severe storms with values 5–10% higher. Some coastal regions in the SH extratropical region show mean ${H}_{s}$ during storms of similar order of magnitude, such as the southernmost coast of Chile, southern New Zealand and some parts of the southern Australian coast.

The coasts affected by TCs show mean ${H}_{s}$ consistently higher than 2 m. Furthermore, these coasts show a distinct increase in mean ${H}_{s}$ for severe storms with respect to regular storms, exceeding 25% in, for example, the southern coasts of Japan and the northwestern coast of Australia. The high mean ${H}_{s}$ of the storms along the coasts of the Arabian Sea during the summer monsoon, with mean ${H}_{s}$ close to 3.5 m, are noteworthy, which when combined with the extremely long storm durations (Fig. 1b), significantly increases destructive power. Open coastlines directly affected by the propagation of energetic swells from the extratropical regions, such as the south coast of Sumatra, southern India and Sri Lanka, the coasts of Chile and Peru and the Atlantic coast of Morocco (among others), also suffer wave storms with notably high wave heights (> 2 m), together with very long periods (Fig. SM19; 5–95th ${T}_{m}$ percentiles in Fig. SM21c-d).

The lowest wave storm mean ${H}_{s}$ values can be observed in some marginal seas in intertropical and subtropical latitudes (e.g., the Persian Gulf, Gulf of Thailand) and in the coastlines facing the interior seas in Indonesia (Java and Banda Seas). The mean ${H}_{s}$ of storms in these regions range only between 0.5 to 2 m. Although in general these coasts show moderate increases lower than 10% in mean ${H}_{s}$ of severe wave storms relative to regular storms, the north coast of Java stands out with a significant increase exceeding 20%.

Note that the most extreme values are smoothed in the ${H}_{s}$ averaging during the storm event (hourly resolution). Therefore, in order to appreciate the most extreme wave storms reaching the global coastlines, Fig. SM17 shows the mean of the maximum ${H}_{s}$ in extreme storms in each of the 42 years analyzed (i.e., the mean annual maxima ${H}_{s}$). Results show values higher than 10 m in the northwestern European coasts and the southernmost part of the Chilean coast. In addition, the TC activity is more clearly reflected by this parameter, with ${H}_{s}$ values exceeding 9 m in the NH tropical western Pacific Ocean, 6 m in the SH tropical west Indian Ocean and 4 m in the Gulf of Mexico and Caribbean Sea. For completeness, the mean storm-peak ${H}_{s}$ during wave storms and severe wave storms are included in Fig. SM18.

The mean ${\theta }_{m}$ (Eq. 6) of wave storms (Fig. 4c) shows that the wave storm climate along the western coasts of the continents is mostly driven by waves generated by extratropical storms. Thus, the mean ${\theta }_{m}$ during storms along these coasts is mainly determined by the propagating trajectory of these wave systems (e.g., mean ${\theta }_{m}$ in northwestern Europe is westerly and in South Australia it is southwesterly).

To a very good extent, wave storm climate in the eastern coastlines of the continents is more complex than in the western coastlines. This is due to the coexistence of wave storms with different geneses across the year (ETCs or TCs, for example).Additionally, the trade winds are the main driving force of the wave storm climate in the eastern intertropical and subtropical coastlines, inducing mean ${\theta }_{m}$ during storms with a prominent east component. Also, most of the eastern tropical coastal areas are affected by TCs. Therefore, ETCs, trade winds and TCs are the main wave storm generating systems impacting the eastern continental coasts, apart from local systems such as the Nortes-induced wave storms in the Gulf of Mexico, or the Monsoon-induced wave storms in the Arabian Sea.

Results for severe wave storms are very similar to those for wave storms, as the differences are lower than 15º in 85% of the global coastlines (Fig. 4d). Most of the exceptions show that severe wave storms are significantly rotated anticlockwise compared to regular wave storms, (e.g., Atlantic coast of USA,the east coast of India or the southwest coast of Spain). Others, such as the northwest coast of Australia or the east coast of Taiwan show the opposite behavior, i.e. clockwise rotation of severe wave storms with respect to regular wave storms.

Besides the integrated parameters included in Fig. 4, the mean ${E}_{f}$ (see “Methods” for ${E}_{f}$ definition) during wave storms has also been assessed (Fig. SM20; 5–95th ${E}_{f}$ percentiles in Fig. SM21e-f). The highest ${E}_{f}$ values are observed along the coastlines affected by storms with the highest mean ${H}_{s}$, and at the same time characterized by long ${T}_{m}$. On the other hand, the coasts exposed to swell storms with the longest ${T}_{m}$ and relatively low ${H}_{s}$, such as in the Gulf of Guinea and South Sumatra, do not show energy fluxes as high as those in areas with the highest storm mean ${H}_{s}$. Therefore, the highest ${E}_{f}$ are observed along the western extratropical coastlines, as in the west coast of Ireland and in the southernmost coast of Chile.

#### Wind–sea vs. swell dominance during wave storms

The characteristics of waves can vary significantly based on their stage of development. Therefore, swell storms and wind–sea storms may pose significant differences on the impact they may cause along the coast. For example, the erosive response of a sandy beach to storms will differ depending on whether it is caused by swell waves or wind–sea waves^70,71^. Likewise, the overtopping over coastal defenses caused by waves can also vary according to the swell/wind–sea nature of the waves^72,73^.

**Figure 5:**
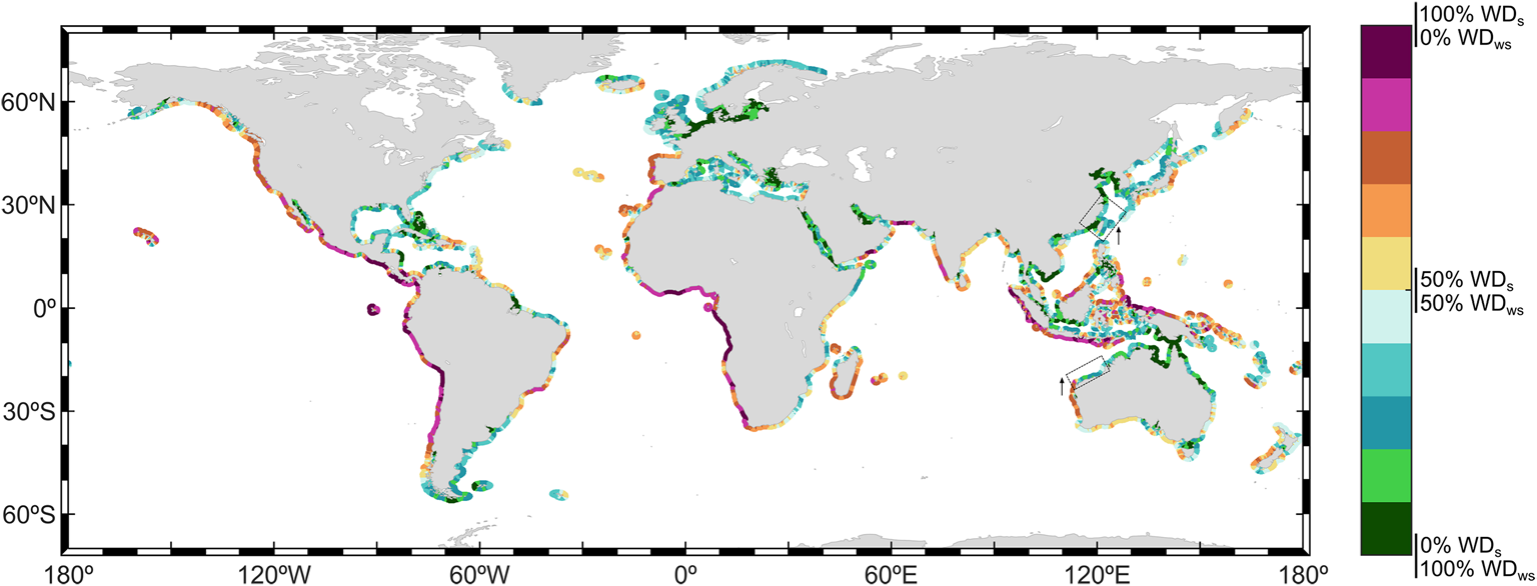
Global coastal mean swell (WD_s_) vs. wind–sea (WD_ws_) dominance during wave storms (in %; Eqs. 8–9). Coastal regions where the difference in the dominance between wave storms and severe wave storms is higher than 10% are highlighted (dashed boxes). Arrows’ direction indicates an increase or a decrease in the swell dominance. The plots were generated using MATLAB R2023b (https://matlab.mathworks.com).

Results show that wave storms are mostly dominated by wind–sea waves (Fig. 5). By definition, a wave storm is a highly convoluted sea state where waves are, in most cases, under their generation process. However, by using a local quantitative approach to define the wave storm threshold, coastal sea states can be classified as storms under close to pure swell sea states if ${H}_{s}$ conditions significantly exceed the mean ${H}_{s}$ climatology (see “Methods”). Therefore, the mean proportion of wind–sea and swell energy during storm sea states will determine the wind–sea vs. swell dominance at a certain coastal location (${WD}_{s}$ and ${WD}_{ws}$, Eqs. 8–9). The proportion of the global coastlines dominated by swells (i.e., ${WD}_{s}$>50%) during wave storms is around 40% and that percentage drops to around 35% if only severe storms are considered.

Differences between the eastern and western open ocean coastlines are noticeable, due to the dominant wave (and atmospheric) eastward propagation paths in extratropical latitudes, as well as due to the predominant cyclogenesis areas of extratropical storms over the ocean. The opposite occurs in lower latitudes, with the trade winds blowing in the opposite direction along with wave systems and wave propagation. For that reason, western coastlines are mainly dominated by swells, especially in the intertropical and subtropical latitudes, due to the propagation of extratropical swells equatorward, reaching swell dominance values during wave storms of above 75% in the coasts of the Gulf of Guinea, for example. As latitude increases, wave storms start to be mostly controlled by wind–sea waves. Poleward of 40º, coasts are impacted by very high waves, mostly still on their developing stage (wind–seas). Additionally, Table SM2 summarizes the main coastal regions showing plain wind–sea and swell dominance patterns during storms.

The northwestern European coasts show the most intense wind–sea dominance pattern during storms, especially in the North Sea, due to the protection against swell storms generated remotely. The extratropical North Pacific storm track shows its highest ETC track density close to the west coast of Alaska and a decrease to the east^74,75^, which is consistent with the wind–sea dominance pattern found. Nevertheless, the fact that western American coasts are, mostly, fully opened to the ocean, makes them susceptible to being impacted by energetic swells generated remotely, thus countering the wind–sea wave storms dominance, most likely explaining the swell dominance in the east coast of the Gulf of Alaska. Finally, regarding the SH, it is worth mentioning that South America reaches the southernmost part of any continent (except for Antarctica), extending poleward of 50ºS. The extratropical southern storm track reaches the coast of Chile, in latitudes characterized by a high-cyclone track density^75,76^, inducing very high wind–sea waves that dominate the wave storm climate there. The strong wind–sea dominance of storms in the southernmost part of the Argentinean coast, a coast protected against the high westerly swells generated in the South Indian Ocean, reflects the strong wind–sea wave storm activity in these latitudes.

On the other hand, the storm dominance along the eastern coastlines of the continents is not so clear, showing an heterogenous pattern combining wind–sea-dominated storm coasts (e.g., northeast coast of Australia), swell-dominated storm coasts (e.g., north coast of Papua New Guinea) and coastal regions showing a split dominance (e.g., north coast of Brazil). Marginal and semi-enclosed seas, protected to the open ocean, show, in general, a clear wind–sea dominance of storms (e.g., Gulf of Mexico, Red Sea).

The dominance pattern for severe wave storms is very similar to the one for regular wave storms. The main exceptions found (highlighted in Fig. 5) show an increase in the swell dominance during severe storms (e.g., east coast of Taiwan, northwest coast of Australia).

#### Storm intensity

**Figure 6:**
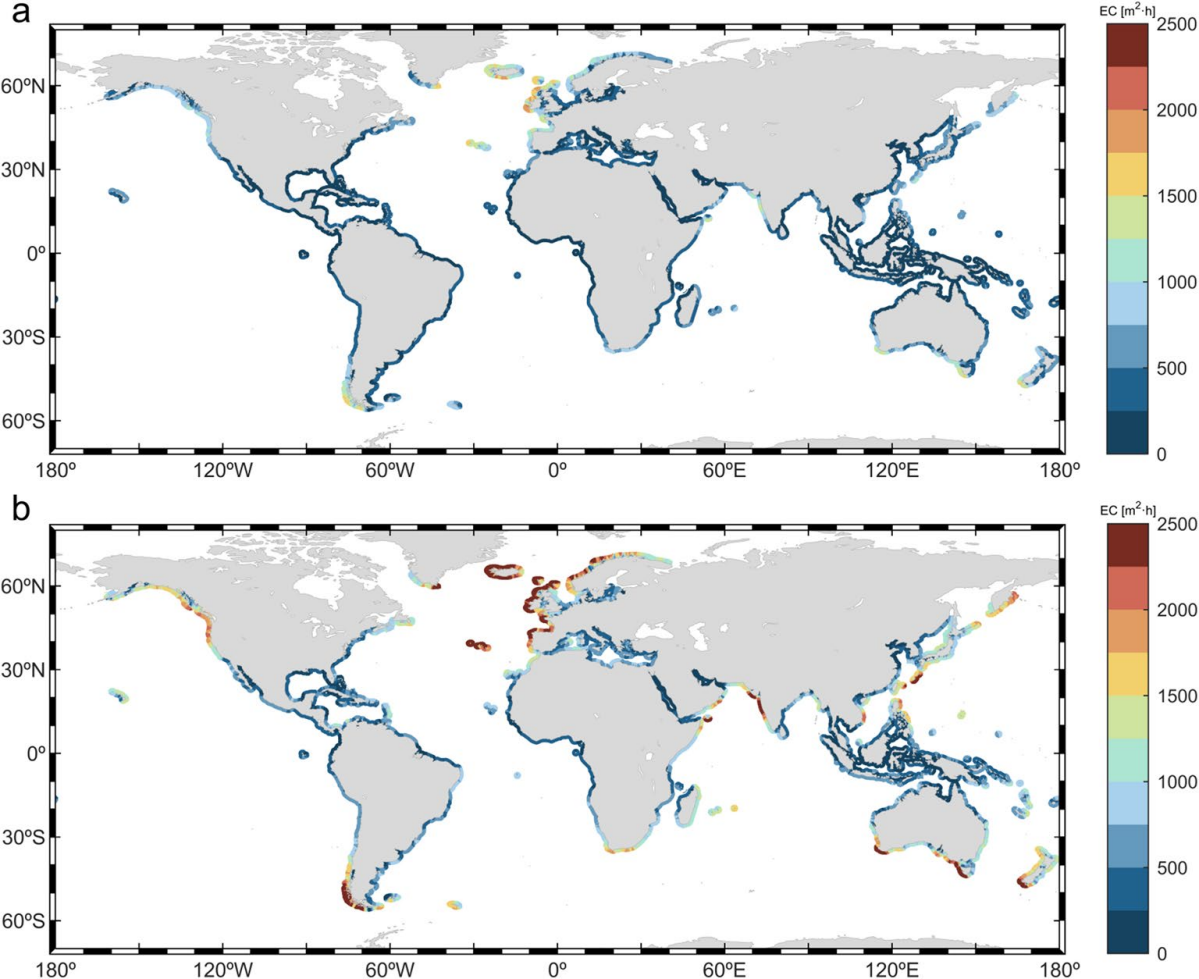
Global coastal mean energy content (EC; in m^2^h) for (**a**) wave storms and (**b**) severe wave storms. The plots were generated using MATLAB R2023b (https://matlab.mathworks.com).

The intensity of storms, quantified as the energy transported by these events, has been related in previous studies to coastal processes such as coastal erosion^53,54,77^. The intensity of wave storms is assessed in the present study through two indices: the energy content (EC, Eq. 1) and the total storm wave energy (TSWE, Eq. 7). The 5–95th percentiles for both intensity metrics are included in Fig. SM23. Despite only TSWE considering the wave period, results show a very similar global intensity pattern with respect to EC index, which is only based on storm wave heights. Note that the fact that both indices include the storm duration (integrating in time) explains the similar patterns along coastlines impacted by very long storms (e.g., Western India). Therefore, only results for EC index will be shown here (Fig. 6), whereas those for TSWE are depicted in Fig. SM22. Additionally, the coastal regions showing the strongest intensities are ranked in Table 3.

**Table 3:** First column: ranking order. Second column: top 5 coastal regions impacted by the most intense wave storms along the global coastlines. Third column: maximum EC index (m^2^h) of wave storms and, between brackets, of severe wave storms within the region. Fourth column: maximum TSWE index (kWh/m) of wave storms and, between brackets, of severe wave storms within the region. k indicates that the value is multiplied by 10^3^. (+) indicates that values could exceed the upper limit of the range.

Rank	Coastal region	EC (m^2^h)	TSWE (kWh/m)
1	Northwestern Europe	1.75–2 k_(2.25–2.5 k+)_	10.5–12 k_(13.5–15 k+)_
2	Southwestern South America	1.5–1.75 k_(2.25–2.5 k+)_	9–10.5 k_(13.5–15 k)_
3	Southern Australia	1.5–1.75 k_(2.25–2.5 k+)_	7.5–9 k_(13.5–15 k+)_
4	Southwestern Europe	1.25–1.5 k_(2.25–2.5 k+)_	7.5–9 k_(13.5–15 k+)_
5	Western India	1.25–1.5 k_(2.25–2.5 k+)_	6–7.5 k_(13.5–15 k)_

Results show a strong gradient between the high storm intensities along extratropical coasts and low intensities in intertropical and subtropical latitudes both for wave storms and severe wave storms (Fig. 6a,b, respectively). In the NH, the northwestern European coast experience the most intense storm events, reaching EC values of 2000 m^2^h and, for severe storms, intensities that can exceed ECs of 3400 m^2^h. Wave storms reaching the coasts in the SH extratropical region are also characterized by high intensities, exceeding 1500 m^2^h for wave storms and 2000 m^2^h for severe storms in the southernmost coasts of Chile, for example. Despite the storm intensity latitudinal gradient, some specific intertropical and subtropical coasts may still suffer the impact of intense wave storms, such as the coastlines along the Arabian Sea during the Indian summer monsoon, mainly due to the very long storm durations (Fig. 1b). The intensity of TCs, which are extremely energetic events, also induces high storm intensities in coastal regions affected by wave storms generated by these events (EC > 1000 m^2^h). The intensity of the storms in the rest of intertropical and subtropical coastlines is much lower, with EC values of around 500 m^2^h or less.

### Degree of coastal storminess classification

**Figure 7:**
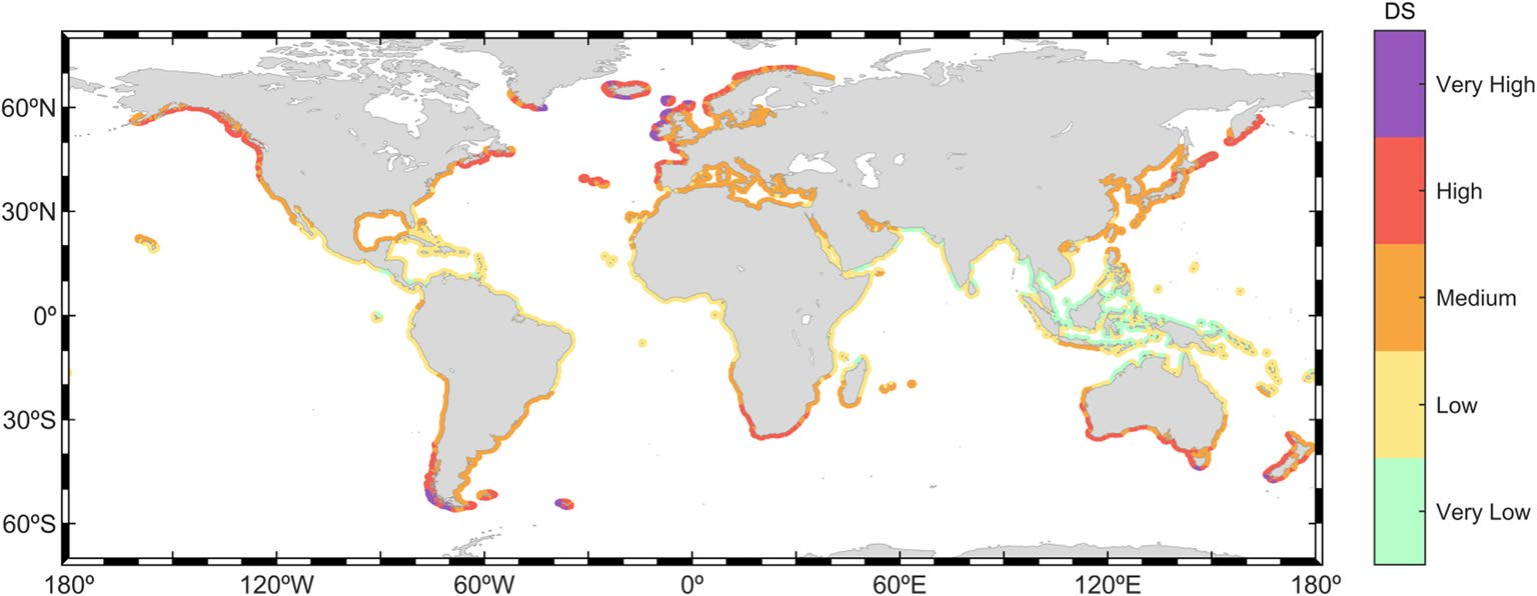
Classification of the global coastlines in terms of the degree of storminess (Eqs. 10–13). The plot was generated using MATLAB R2023b (https://matlab.mathworks.com).

The global coastlines are classified in terms of the wave storminess based on a new index, the degree of storminess. The DS parameter introduced here (Eqs. 10–13) provides an integrated qualitative view of how stormy a coast can be (Fig. 7). The integration of the number of events blended with the intensity index (EC, Eq. 1) offers information about the duration and the wave height of the events. Results show, a clear latitudinal gradient, albeit with some exceptions.

Only a few coastal areas are classified as very high DS. These coasts are, in the NH, the west coasts of Ireland and Scotland, the westernmost coast of the Azores Islands and the south coasts of Iceland and Greenland. In the SH, Tasmania and the southernmost coast of Chile are also classified as very high DS. These regions are characterized by their prone geographical location, at the extratropical latitudes of both hemispheres, and exposed to vast areas of open ocean. They are highly energetic areas, frequently experiencing intense storms, a situation favored by long fetches and strong atmospheric-generating conditions. The coasts classified with a high DS are always poleward of 30º (e.g., Gulf of Alaska, Southern Australia). The remainder of coasts poleward of 30º can be broadly classified as medium stormy.

In the intertropical and subtropical regions, coastlines are characterized by low or very low DS. Exceptions (medium DS) occur in the western latitudinal coasts of the South Pacific Ocean, affected by cyclones, such as the south coasts of Japan, Taiwan and the north shores of the Philippines. The coast of the Gulf of Mexico (medium DS), mainly affected by wave storms caused by the *Nortes* systems and by TCs wave storms, is also an exception. A very low storminess can be found in intertropical latitude coasts, close to the equator, in all oceans. Thus, the main coasts falling into very low DS classification are the protected coasts of Indonesia, Northern Papua-New Guinea, Northern Australia, Southwestern India and the southernmost coasts of Myanmar. In general, these regions experience storms with low wave heights due to their geographical sheltering from the paths of extreme waves originating in the open ocean, particularly in the Indian and Pacific Oceans. Furthermore, within the internal seas of the Maritime Continent, wave growth is constrained by the short fetches. Consequently, the storm intensity in these areas is very low, which in turn results in a very low DS.

### Storm wave climate classification

**Figure 8:**
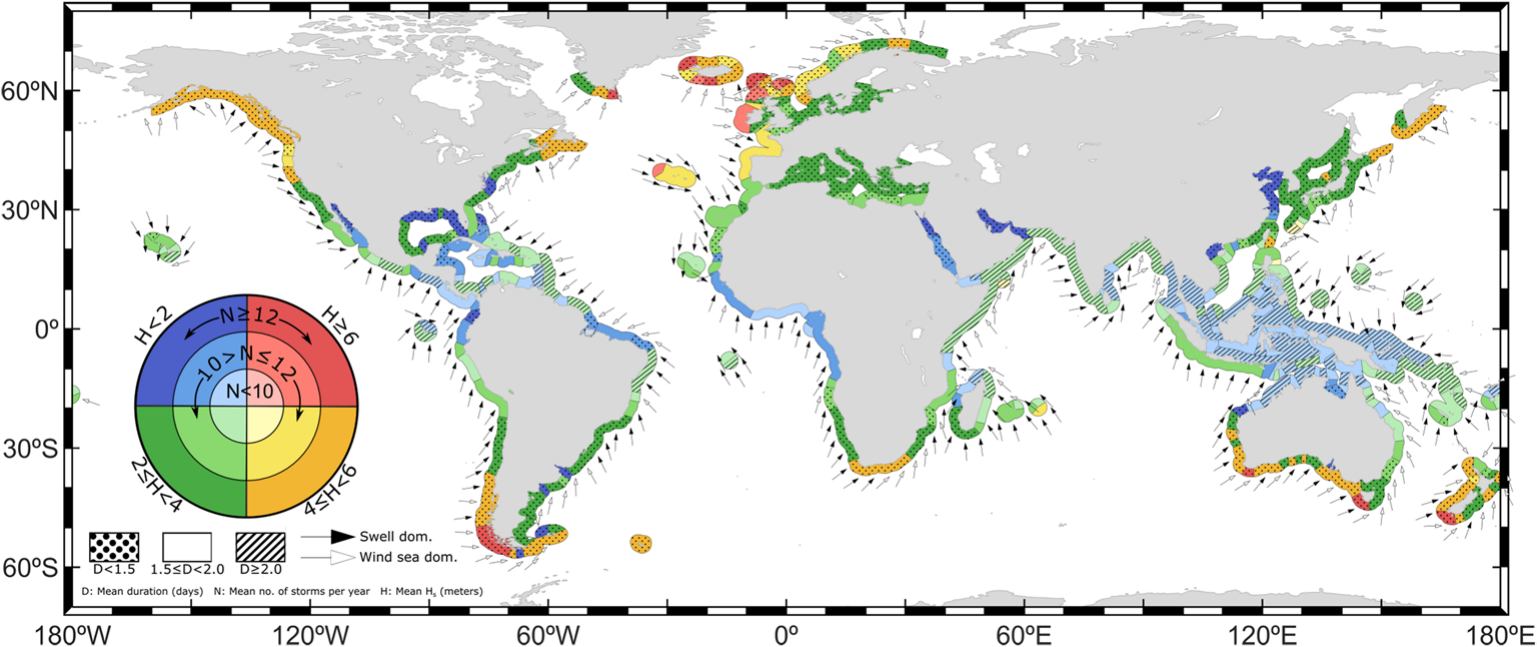
Storm wave climate classification along the global coastlines according to key characteristics of wave storms: mean *H*_*s*_ (color), mean number of events (color scale), mean storm duration (hatching), mean ${\theta }_{m}$ (arrow direction) and swell vs. wind–sea dominance (arrowhead color). The plot was generated using MATLAB R2023b (https://matlab.mathworks.com).

Figure 8 provides a holistic classification that integrates the main individual characteristics of storm events: frequency of occurrence, duration, wave height, direction and the swell vs. wind–sea dominance during storm events. Each storm characteristic is divided into different ranges of variation and represented differently so that all can be displayed together.

The information depicted in Fig. 8 allows an easy identification of coastlines, sometimes of hundreds of kilometers, which have a similar wave storm climate. For example, the coasts along the Gulf of Guinea, which extends for more than 3000 km, shows a homogenous wave storm climate with mean ${H}_{s}$ lower than 2 m and mean duration between 1.5 and 2.5 days. In addition, it makes the comparison between the wave storm conditions in coasts from different regions and continents easier. It is possible to see, for example, that the main characteristics of the wave storms at the southernmost coast of Chile and the west coast of Tasmania are quite similar in terms of mean duration, mean ${H}_{s}$ and mean frequency. The same occurs for the Gulf of Alaska and South South-Africa. In this regard, it is noteworthy to point out that all the coasts classified with a very high degree of storminess share relatively high frequency of occurrence of wave storms, more than 10 times per year, as well as very high wave heights (above 6 m), with mean durations of less than 1.5 days. Contrarily, coasts classified with very low storminess are affected by storms of low wave heights, normally below 2 m, with longer durations: more than 1.5 days.

## Discussion and conclusions

The present study has assessed the wave storm characteristics along the global coastlines, based on the output of a 42-year global wave hindcast at a relative high resolution (16 km). Despite multiple storm classification criteria used in previous studies, for simplicity and consistency across the global coastlines, a unique criterion widely used in the literature has been applied: sea state events are classified as wave storms by analyzing the exceedances over the ${H}_{s}$ 95th percentile threshold. Additionally, the most energetic wave storms (severe wave storms), are isolated based on their energy content and independently analyzed.

Some wave storm statistics and indices are presented here for the first time, such as inter-arrival time between storm events within storm seasons and the degree of storminess (see “Methods”). The key features of wave storms: frequency of occurrence, duration and intensity, as well as the mean integrated wave parameters ${H}_{s}$ and ${\theta }_{m}$, have been analyzed. For completeness, the swell vs. wind–sea dominance during the storm events has also been assessed.

A classification of coastal wave storminess, based on the new index DS (Fig. 7), is proposed. This new metric combines the energy content and the frequency of occurrence of wave storms. The coastal storminess has been categorized considering solely the energy of the events in multiple studies, frequently considering the energy content as the basis for these classifications^54,55^. The integration of the energy of the storms with the number of storms impacting the coastlines acknowledges that both factors play crucial roles in assessing coastal impacts. A higher frequency of events would potentially suggest a greater number of impact events, whereas a higher energy content would likely imply greater severity of the impacts. Since DS is calculated by adding both metrics with an even contribution, it can be deduced that a very high DS corresponds to high values of both characteristics. Therefore, coastal regions affected by a very high DS are potentially exposed to more frequent and severe impact events. Similarly, coastal regions classified with a high DS may experience medium-to-high frequency of impactful events with corresponding medium-to-high severity.

The storminess pattern along global coastlines is shown to be characterized by a latitudinal gradient, with higher DS at coastlines in higher latitudes in both hemispheres. The coasts in the extra-tropical regions experience a higher mean number of storm events per year, each of them lasting, on average, less than 1.5 days. These storms are generated by ETCs, with a higher frequency of occurrence in the respective hemisphere winter and a strong seasonality. Wave storms arriving at the coast with a certain incoming direction can either be generated remotely and propagate as swells, or they can occur with the landfall of a storm, then dominated by wind–seas. Coasts in extratropical latitudes, especially in the western continental coastlines, are impacted by highly intense storms with the highest mean ${H}_{s}$ along the global coastlines, exceeding 6 m in some coastal regions. Poleward of 40º, in both hemispheres, wave storms are dominated, in most cases, by wind–sea waves. As latitude reduces, the wind–sea dominance in the western coasts evolves to an almost-full swell dominance and lower mean wave storm ${H}_{s}$. Severe storms in the extra-tropical regions show lower frequencies and, as expected, longer durations, higher mean ${H}_{s}$ and higher intensities.

Wave storms along the intertropical and subtropical coastlines have a more complex wave storm climate due to the convergence of wave storms with different geneses: extra-tropical storms propagating equatorward, wave storms generated by the trade winds, wave storms generated by TCs and wave storms generated by local wind features (e.g., *Nortes* in the Gulf of Mexico or the monsoon in the Arabian Sea). Wave storms generated by ETCs mainly affect the intertropical and subtropical western coasts of the continents. These wave storms are swell-predominant, mostly impacting during the winter months. Trade winds generate low-intensity storms that control the wave storm events along the eastern coasts of the continents in tropical latitudes, in both hemispheres. Wave storms induced by TCs are characterized by high wave heights, and, not surprisingly, dominated by wind–sea waves. Such events can be best observed in the NH tropical western Pacific coasts as this is the region with the highest frequency of TCs in the world^78^, exceeding mean ${H}_{s}$ of 4 m and, if only considering severe storms, 5 m.

However, results in areas affected by TCs should be critically evaluated. Despite the hindcast having been demonstrated to represent storms generated by TCs, an underestimation in the highest peaks can still be observed (Fig. SM7). Moreover, despite the approach used to define storms having been used in TCs regions in previous studies^47^, it could lead to including wave events with different origins than TCs, thus masking, in part, the extreme values associated with these events. An additional analysis of the mean annual maximum ${H}_{s}$ helps to better identify the trace of TCs along the coastline, showing values of 8 m in the NH tropical western Pacific coasts (Fig. SM17).

This study also presents a series of limitations that need to be addressed for a critical interpretation of the results. Regarding the data used, a single hindcast product has been utilized. In this regard, recent studies have shown notable differences between hindcast products, particularly in the representation of extremes^79^. However, the validation conducted for the present hindcast (Fig. SM1-SM7) indicates the robustness of our results. Moreover, results have been compared against another hindcast (GOW2^80^). Despite this product being developed using a different numerical propagation model (WAM vs. WW3) and driver forcings (ERA5 vs. CFSR), results show good consistency between both products (Fig. SM25). Nevertheless, results of this study should be interpreted with the awareness that the hindcast product do not provide perfect results and that slight discrepancies could be found when utilizing a different historical wave database.

It is also necessary to mention that this study was conducted using a standard simplified methodology to facilitate valid comparisons among different coastal regions. However, the chosen methodology for defining wave storms also has inherent limitations. To facilitate a comprehensive global assessment and due to computational constraints linked to the scale of the study, certain simplifications were made. Therefore, when applying these findings to inform high-resolution local coastal impact studies or infrastructure design, a critical assessment is essential. For example, selecting a site-specific threshold for conducting a local scale study is recommended, especially if the findings are intended for subsequent extreme value analysis and return period estimations at the site. Moreover, the use of a 12-h minimum duration criterion may vary depending on the goal of the study, as it can result too long or too short events. If a local study is being conducted on a coastline influenced by a diurnal tidal regime or in an area where tide is not a determining factor for extreme water level occurrences, different criteria might be needed. The same applies for the independence time between storms. While statistical analyses in this study generally ensure storm independence, a local study could potentially achieve this with a shorter period (or even require a longer one). Consequently, while this study provides a standardized and consistent approach to global wave storminess and efforts have been made to integrate the global results, readers must be aware of the underlying assumptions and the local scale and case-focus character of the problem.

Nevertheless, despite the existing limitations, this study provides holistic understanding of the main characteristics of coastal wave storms at a global scale, which have been summarized in Fig. 8. The use of a unique and flexible criterion that adopts the local climate and a high enough threshold to classify storms, as well as the isolation and independent analysis of severe storms, provide a reliable basis to understand the global wave storm behavior and illustrates the main differences in storm climate in different coastal regions. The findings presented here could be useful in other oceanography fields, such as fisheries, the offshore energy industry and navigation. Additionally, the they could be used as a reliable source for identifying coastal areas experiencing a high wave storminess, as well as to specifically identify certain storm characteristics affecting certain coastal processes. These insights could be valuable in the development of adaptation strategies, assessment of environmental and ecological impacts of storms, coastal development and land use planning, or coastal management and disaster preparedness.

## Methods

### Wave climate data

The present study uses a global high-resolution wave hindcast, produced by the ECMWF, covering the period from 1979 to 2020, and produced using ERA-5 reanalysis forcing and sea ice coverage. The ERA-5 is the latest ECMWF global coupled atmosphere-wave reanalysis^57^. Its atmospheric data have a horizontal resolution of 32 km (TL639), whereas its ocean wave products have a resolution of 40 km (0.36°; 24 directions and 30 frequencies), both are available with the time resolution of 1 h^57^.

A high-resolution (16 km, Tco639, horizontal resolution and hourly output) stand-alone run (not coupled) global wave hindcast, forced with hourly ERA-5 neutral 10 m winds, air density, wind gustiness and sea ice coverage, has been produced: the ERA-5H. It was obtained using a more recent version of the ECMWF IFS (Integrated Forecasting System Cy46r1; ECMWF 2019), with an improved wave physics for wind input and swell dissipation^81^, a more recent global bathymetry (ETOPO1^82^) and a finer spectral resolution (36 directions and 36 frequencies). No ocean surface currents or changing water level were considered.

The current study uses hourly wave fields of ${H}_{s}$, mean wave energy period (${T}_{m}$), and mean wave direction (${\theta }_{m}$) parameters from ERA-5H. Grid nodes close to the global coastal areas along the coastline were selected to conduct the analysis. In particular, the second closest points to the coasts were selected for the analysis, which secures that most points are located at depths (Fig. SM8) associated with deep or intermediate waters. An analysis of the depth-induced wave breaking at these points indicates that less of 0.1% of the analyzed points could experience this phenomenon, even for the largest waves (see Supplementary Material). Additionally, to ensure sufficient data during stormy seasons, grid nodes covered by ice for more than 15% of the time within winter months are not considered in the analysis^83^.

A validation and performance evaluation of the hindcast, particularly focused on coastal regions, is presented in Supplementary Material based on the comparison with in-situ observations. A total of 286 in-situ locations have been selected for validation purposes. Results show a good performance of the hindcast in the representation of the parameter *H*_*s*_, a pivotal element of the selected approach (Fig. SM1-SM3). Additionally, the representation of extremes, analyzed through the 95th percentile *H*_*s*_, calculated over the available buoy data period, also show good results against instrumental data (Fig. SM4-SM6).

A specific validation of the representation of TCs has been conducted in the Gulf of Mexico and the southern coast of Japan (Fig. SM7). Results demonstrate that the TCs are reasonably well represented within the model outcomes. Nevertheless, a slight underestimation can be seen in the most extreme wave height peaks.

A detailed analysis at twenty-four specific locations (key points) distributed across the global coastal regions has also been conducted. The points were selected based on two primary factors. The first was to ensure a homogeneous coverage of the global coastlines, and the second was to represent the diverse storm conditions.

Regarding the first factor, ten of these points are located along the Pacific Ocean coastlines, five of them in the Northern Hemisphere (NH) and five in the Southern Hemisphere (SH). Eight points are disposed along the Atlantic Ocean coastlines, five in the NH and three in the SH. One more point is in the Mediterranean Sea. The remaining five points are in the Indian Ocean coastlines, two in the NH and three in the SH.

With respect to the second factor, the coastal regions experiencing the highest number of events are represented by P3, P5, P7 and P24. For example, the longest durations, which are found in the Arabian Sea, are represented by point P11. The highest wave heights are captured by P4, P20 and P22, while the longest periods are represented by P14, P16, P18 and P22. The effects of TC-induced waves are represented at points P7, P13 and P17.

### Wave storm definition

The ${H}_{s}$ 95th percentile (${H}_{s}95$) at a specific coastal position is selected as threshold to classify wave events as wave storms, following similar approaches as previous studies at regional scale^32,41–43,84^. This method has been chosen as it provides a different magnitude for the threshold based on the local wave conditions^85^, which is an essential requirement for large-scale assessments (global in this case). This is in contrast to a single absolute magnitude threshold (e.g., 3 m), which does not offer the same level of adaptability.

The wave storm classification also requires two additional criteria: the calm sea state period between consecutive storms and the minimum duration of storm events^86^. The time between storms must ensure the independence between consecutive events. Hence, two wave storm events are here considered as independent when the time between the two consecutive ${H}_{s}$ peaks over the ${H}_{s}95$ threshold exceeds 48 hours^87,88^. The validity of this assumption has been tested using Kendall’s tau correlation parameter^89^, as it has been done in previous studies^90,91^. Results demonstrate that events can be considered as independent in most of the global coastlines (> 85% at 5% significance level; Fig. SM24).

In this study, the duration of the storm is defined as the exceedance time over the ${H}_{s}95$ threshold between the starting time and the end of the event. Imposing a minimum duration of the storms avoids short sparse exceedances that would not have a significant impact on the coast. In fact, besides the energy impairing the coastline, i.e., the intensity of the wave storm, the duration of that extreme event is in fact what causes more impact on the coast^92^. The storm duration criterion has been chosen in previous studies according to different criteria, such as the time series resolution^32^, the duration of the tidal cycle^52,84,93,94^, or the minimum duration assumed to cause erosion damage in the coastline^31,53,95^. Here, we choose a single minimum storm duration of 12 h, for several reasons. First, the use of a single criterion is the standard practice in regional studies^42,47,50,96^ and it allows a homogenous comparison across coastal areas. Second, the selected minimum duration has been used in multiple studies, covering coastal regions affected by different storm wave climates, which encourage its use at global scale: Mediterranean Sea^50,96–99^, the Gulf of Mexico and the Caribbean Sea^47^, Eastern North Atlantic^31,52,84,93,95,99,100^, Western North Atlantic^88,100^, the Eastern North Pacific^94,99^ and the Black Sea^101^. Third, this criterion captures long events, avoiding sparse weak events that may not cause coastal damage. Fourth, most common values of minimum duration in the literature are 6 h and 12 h^86^, so the selected value is within the given range. Lastly, this criterion ensures that wave storms occur during at least one tidal cycle in semi-diurnal and mixed tide regimes, which cover most of the global coastlines^102,103^.

Following the criteria mentioned above, in summary:

- a wave storm event starts at the up-crossing time and it ends at the down-crossing time of the ${H}_{s}95$ threshold;
- when the time between these two moments is higher than 12 h; and
- two wave storms are considered independent, and classified as two single events, when the time between the ${H}_{s}$ peaks is higher than 48 h (if the time gap is lower than that the two events are merged into one wave storm).

Nevertheless, since this approach may provide too weak storms in some coastal areas, smoothing out relevant information about extreme sea state events, severe wave storms are identified by assessing the intensity of the storms. The intensity of the storms is quantified through the energy content index ($EC$)^53^, which is calculated as the integration of ${H}_{s}^{2}$ during storm events:

1 $$EC=\frac{{\sum }_{i=1}^{i={N}_{t}}{\int }_{{t}_{0,i}}^{{t}_{f,i}}{H}_{s}^{2}dt}{{N}_{t}},$$

where ${t}_{0,i}$ is the starting time of the storm $i$, ${t}_{f,i}$ is the ending time of the storm $i$ and ${N}_{t}$ is the total number of storms.

Thus, after computing the *EC* of wave storms, severe wave storms are defined as those events exceeding the upper quartile of energy—in other words, storms exceeding the 75^th^ percentile *EC*, which ensures severe storms to be highly energetic events and potentially more impacting along the global coastlines.

### Wave storms analysis

The wave storm frequency of occurrence has been computed annually and seasonally, hence resulting in the mean number of storms ($N$) per year or season at each location, respectively, as:

2 $$N=\frac{{N}_{tr}}{{N}_{y}},$$

where ${N}_{tr}$ is the total number of storms over the reference period (i.e., year, season or month), calculated over the total hindcast period, and ${N}_{y}$ is the number of available hindcast years.

The number of storm seasons, i.e. the continuous periods of time recurrently affected by the impact of wave storms, is also assessed. This number is computed by first estimating the minimum number of months where at least 75% of the storms occur (${M}_{75}$) and, then, the number of independent periods in which they group (i.e., periods separated by at least one month). Along some coasts the monthly number of storms remains more or less constant throughout the year. Since these cases cannot be directly inferred by this method, a new metric that measures the variability of the monthly mean number of storms is proposed (${R}_{m}$), computed as:

3 $${R}_{m}=\frac{{N}_{m, min}}{{N}_{m, max}},$$

where ${N}_{m, min}$ is the minimum monthly mean number of storms and ${N}_{m, max}$ is the maximum monthly mean number of storms.

A value of ${R}_{m}$ < 1/3 may be taken to be indicative of coastal locations with practically no storm seasons.

Because the time between storms is of critical importance for beach recovery after storm events, the mean time between storms within storm seasons is also computed (${IT}_{s}$) at each coastal location, as:

4 $${IT}_{s}=\frac{{\sum }_{k=1}^{k=NS}{\sum }_{i=1}^{i={N}_{s,k}-1}{it}_{s,i,k}}{{N}_{st}-NSe},$$

where ${it}_{s,i,k}$ is the time between the pair $i$ of consecutive storm peaks in storm season $k$, ${N}_{s,k}$ is the total number of storms in the storm season $k$, ${N}_{st}$ is the total number of storms in all storm seasons and $NSe$ is the number of storm seasons.

The mean wave storm duration ($D$) is computed as:

5 $$D=\frac{{\sum }_{i=1}^{i={N}_{t}}{d}_{i}}{{N}_{t}},$$

where ${d}_{i}$ is the duration of each individual storm.

The mean integrated wave parameters of wave storms ($IWP$) are computed as:

6 $$IWP=\frac{{\sum }_{i=1}^{{i=N}_{t}}\overline{{IWP }_{i}}}{{N}_{t}},$$

where $\overline{{IWP }_{i}}$ is the mean integrated wave parameter (e.g., ${H}_{s}, {\theta }_{m}$) during the storm $i$.

The intensity of the wave storms is assessed through two indices. First, the *EC* index^54^. Second, the total storm wave energy^50^ ($TSWE$), calculated as the integration of the wave energy flux $({E}_{f})$ during storm events, defined as ${E}_{f}={c}_{g}E=\frac{\rho {g}^{2}}{64\pi }{T}_{m}{H}_{s}^{2}$; where ${c}_{g}$ is the group speed, $E$ is the total wave energy, $\rho$ is the water density and $g$ is the gravity acceleration. Therefore, both indices consider not only the energy of the storms, but implicitly also its duration, via time integration. These indices are computed at each computational point (node point) across the global coastal areas, as:

7 $$TSWE=\frac{{\sum }_{i=1}^{i={N}_{t}}{\int }_{{t}_{0,i}}^{{t}_{f,i}}{E}_{f}dt}{{N}_{t}},$$

where ${t}_{0,i}$ is the starting time of the storm $i$ and ${t}_{f,i}$ is the ending time of the storm $i$.

The wave field (or sea state) during coastal wave storms can be dominated by wind–sea or swell waves, depending on the respective (wind–sea and swell) energy balance within the wave spectrum of the storm^12^. The wind–sea and swell parts of the wave energy spectrum follows the WAM wave model spectral partition^12,104^, based on the wave age principle^105,106^. The wind–sea and swell dominance ($${WD}_{ws}$$ and $${WD}_{s}$$ , respectively) is calculated based on the ratio between the swell zeroth-order moment ($${m}_{0}^{s}$$) and the total zeroth-order moment ($${m}_{0})$$, as:

8 $${WD}_{s}=\frac{{\sum }_{i=1}^{i={N}_{t}}\stackrel{-}{{\left(\frac{{m}_{0}^{s}}{{m}_{0}}\right)}_{i}}}{{N}_{t}}x100,$$

9 $${WD}_{ws}=100-{WD}_{s},$$

where ${m}_{0}^{s}$ is the swell zeroth order moment and ${m}_{0}$ is the total zeroth order moment.

A new index, named “degree of storminess” ($DS$) is defined in the present study to assess how stormy a coastal location is. It integrates the information about the storm frequency of occurrence, and the storm duration and its significant wave height through the $EC$ index (Eq. 1). DS is computed by attributing the same weight to both $N$ (Eq. 2) and $EC$ (Eq. 1) components.

$N$ and $EC$ are first re-scaled (${I}_{n}$ and ${I}_{ec}$, respectively) by applying the transformation $Y(x)$, as:

10 $${I}_{n}=Y\left(N\right),$$

11 $${I}_{ec}=Y\left(EC\right),$$

where $$Y\left(x\right)=\frac{{x}_{i}-{\text{min}}\left(x\right)}{{\text{max}}\left(x\right)-{\text{min}}\left(x\right)}$$

The degree of storminess ($DS$) is then defined as:

12 $$DS={I}_{n}+{I}_{ec},$$

Finally, $DS$ is re-scaled by applying $Y(x)$ and qualitative labels are attributed as:

13 $$\left\{\begin{array}{c}if\;DS\le 0.2\to Very\; low\\ if \;0.2<DS\le 0.4\to Low\\ if\; 0.4<DS\le 0.6\to Medium\\ if \;0.6<DS\le 0.8\to High\\ DS>0.8\to Very\; high\end{array}\right.$$

The analysis of wave storms is pursued yearly and seasonally. Since the present study is done at a global scale, no distinction is made between (boreal or austral) winter or summer seasons, unless that is specifically needed. Seasons are defined following the WMO (World Meteorological Organization) standards, as: December to February (DJF), March to May (MAM), June to August (JJA), and September to November (SON).

For the sake of coherence, to facilitate the understanding of the geographical coastline locations, in the present study, in both hemispheres, intertropical latitudes are taken to extend from the equator to the tropics, subtropical to extend from the tropics to 35° and extratropical latitudes are higher than 35°.

### Supplementary Information

Supplementary Information.

## Data Availability

The data to conduct the analysis is available at: 10.21957/y03s-tz09, 10.21957/strn-cs36, 10.21957/dgkx-1485, 10.21957/t3vj-b111.
