# Supplementary material for: Global coastal wave storminess
Source: Sci Rep. 2024 Feb 14;14:3726. doi: 10.1038/s41598-024-51420-0 (PMC10866887; doi:10.1038/s41598-024-51420-0)
Supplement: Supplementary file 1 — Supplementary Information. [file 41598_2024_51420_MOESM1_ESM.pdf]

Global coastal wave storminess

Hector Lobeto[1*], Alvaro Semedo[2,3], Gil Lemos[2,3], Ali Dastgheib[2,4], Melisa Menendez[1], Roshanka Ranasinghe[2,5,6], Jean-Raymond Bidlot[7]

[1]IHCantabria - Instituto de Hidráulica Ambiental de la Universidad de Cantabria, Santander, Spain.
[2]Department of Coastal and Urban Risk & Resilience, IHE Delft Institute for Water Education, Delft, Netherlands.
[3]Instituto Dom Luiz (IDL), Faculdade de Ciências, Universidade de Lisboa, Lisboa, Portugal.
[4]IMDC (international marine and dredging company), Antwerp, Belgium.
[5]Department of Infrastructure Engineering, University of Melbourne, Melbourne, Australia.
[6]Department of Resilient Ports and Coasts, Deltares, Delft, Netherlands.
[7]European Centre for Medium-range Weather Forecasts, Reading, UK.

Hindcast validation

The validation focuses on the coastal wave climate. Accordingly, it has been carried out using coastal in-situ observations (from buoys and platforms) as reference. A pseudo-global in-situ dataset consisting of 281 locations was used for validation and performance evaluation, following a selection process (Figure SM1 – SM3). In-situ data was collected and provided by the Copernicus Environment Monitoring Service (CMEMS; Reference: INSITU_NWS_NRT_OBSERVATIONS_013_036), incorporating measurements from recording instruments managed by various institutions. After removing outliers and excluding buoys with anomalous values, two primary criteria were applied in the selection of the buoys for validation:

- The buoy must be located at least 5 km from the coast.
- The buoy must have at least 3 years of recorded data.

Only exceptions are the buoys located in the west coast of India and the coast of Brazil, for which the record length criterion has been relaxed to provide a better coverage of the global coastlines.

Results (panel a of Figure SM1 – SM3) show correlations above 0.9 in around 90% of the in-situ locations analyzed. This indicates a very good agreement between both datasets and highlights the capability of the hindcast to reproduce the wave climate variability at global scale.

Panel b of Figure SM1 – SM3 shows the normalized Hs bias relative to in-situ data. Results show absolute biases lower than 10% at round 80% of the locations. In addition, at around 70% of in-situ locations show the hindcast product shows underestimations. Main exceptions are found in the Gulf of Mexico and Caribbean Sea, and in the southwestern coast of Australia, where a significant proportion of the locations show a positive bias (i.e., overestimation).

Panel c of Figure SM1 – SM3 shows the normalized root-mean square error in the Hs parameter between model and in-situ data. Most locations (~75%) exhibit errors below 20%. The largest errors can be found in the Mediterranean Sea and the northeastern coast of North America, where values typically range between 20% and 30%.

Since the storm definition criterion (see Methods in the main manuscript) is based on the exceedances over the 95th percentile of H_s, a specific validation for this metric, calculated over the available buoy data period, has been conducted (Figure SM4-SM6). Results show a good agreement between the model and the buoy data for H_s95. Most locations (83%) show an underestimation of model data. This underestimation is lower than 10% of the reference value in more than 70% of the analyzed locations. Main exceptions (i.e., overestimations) are found along the western coast of the British Islands and the North Sea, with the latter showing values up to 20%.

In general, results show a good performance of the hindcast product across the world's coastlines, in comparison with observations, providing the necessary confidence to the subsequent storm analysis conducted.

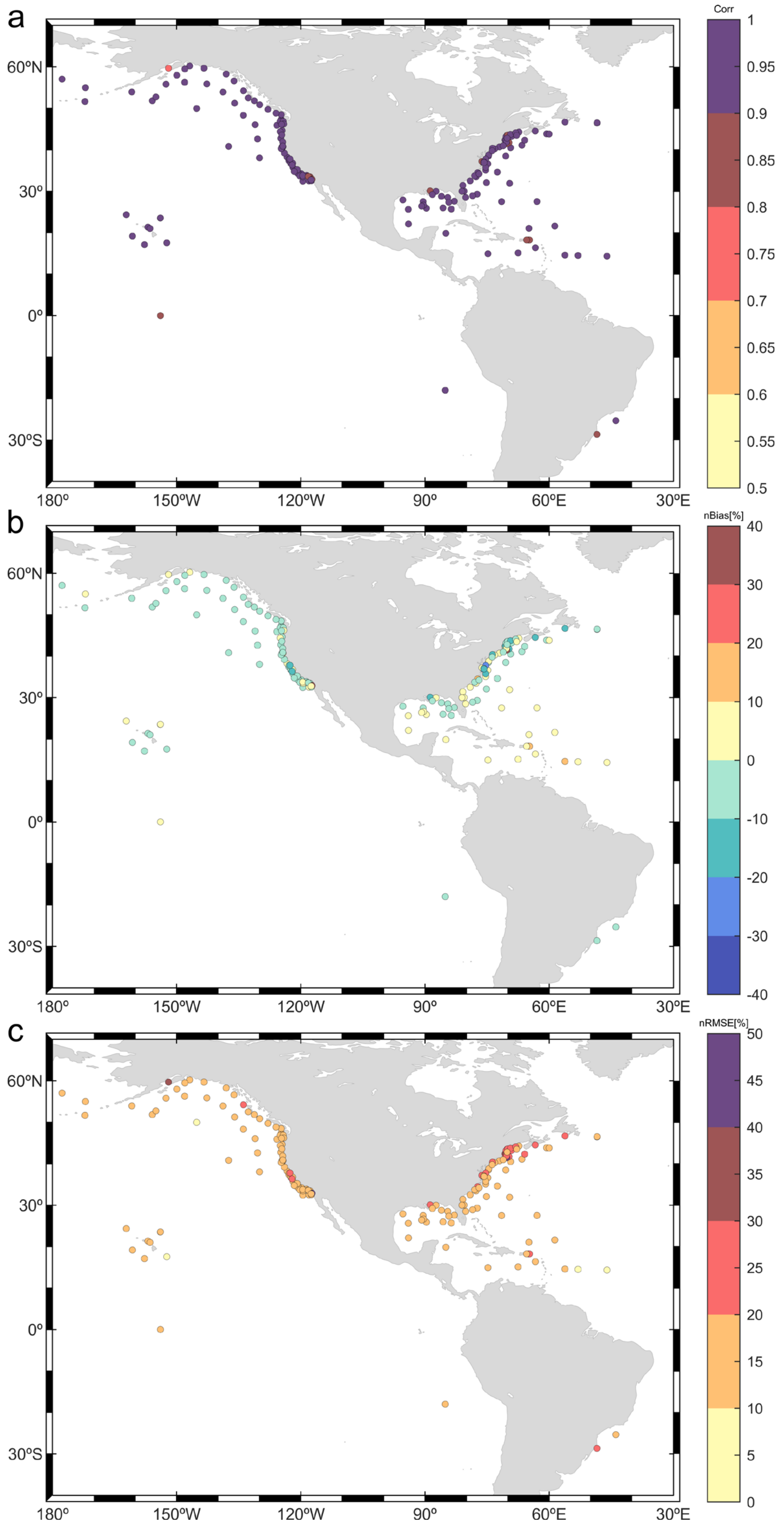

Figure SM1 *Validation of hindcast H_s against buoy data along the coasts of America. (a) Correlation values. (b) Normalized bias (in %) of the H_s parameter from the hindcast product relative to buoy measurements. (c) Normalized root-mean square error (in %) of the H_s parameter from the hindcast product relative to buoy measurements. The plots were generated using MATLAB R2023b (https://matlab.mathworks.com).*

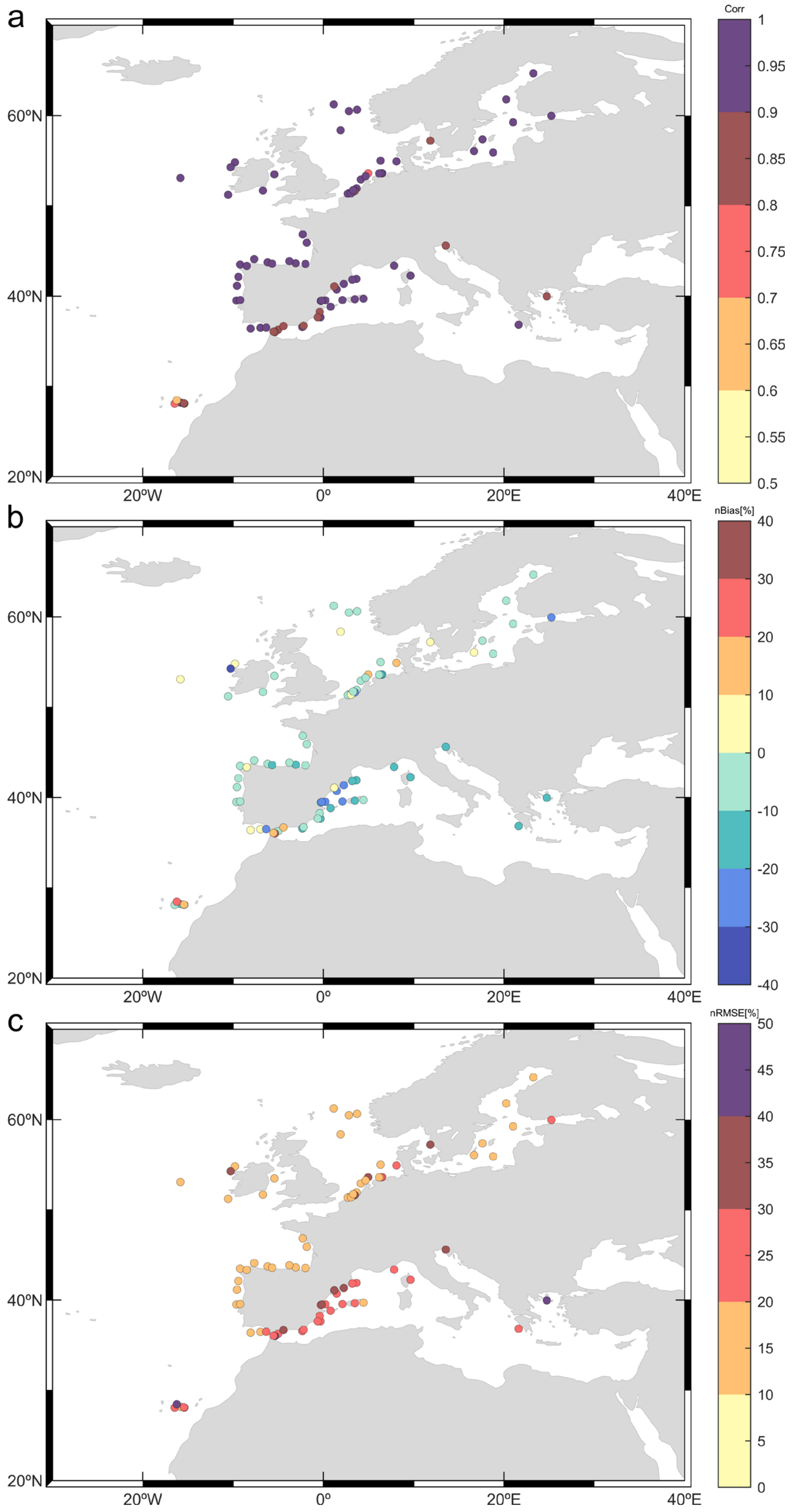

Figure SM2: *Validation of hindcast H_s against buoy data along the coasts of Europe. (a) Correlation values. (b) Normalized bias (in %) of the H_s parameter from the hindcast product relative to buoy measurements. (c) Normalized root-mean square error (in %) of the H_s parameter from the hindcast product relative to buoy measurements. The plots were generated using MATLAB R2023b (https://matlab.mathworks.com).*

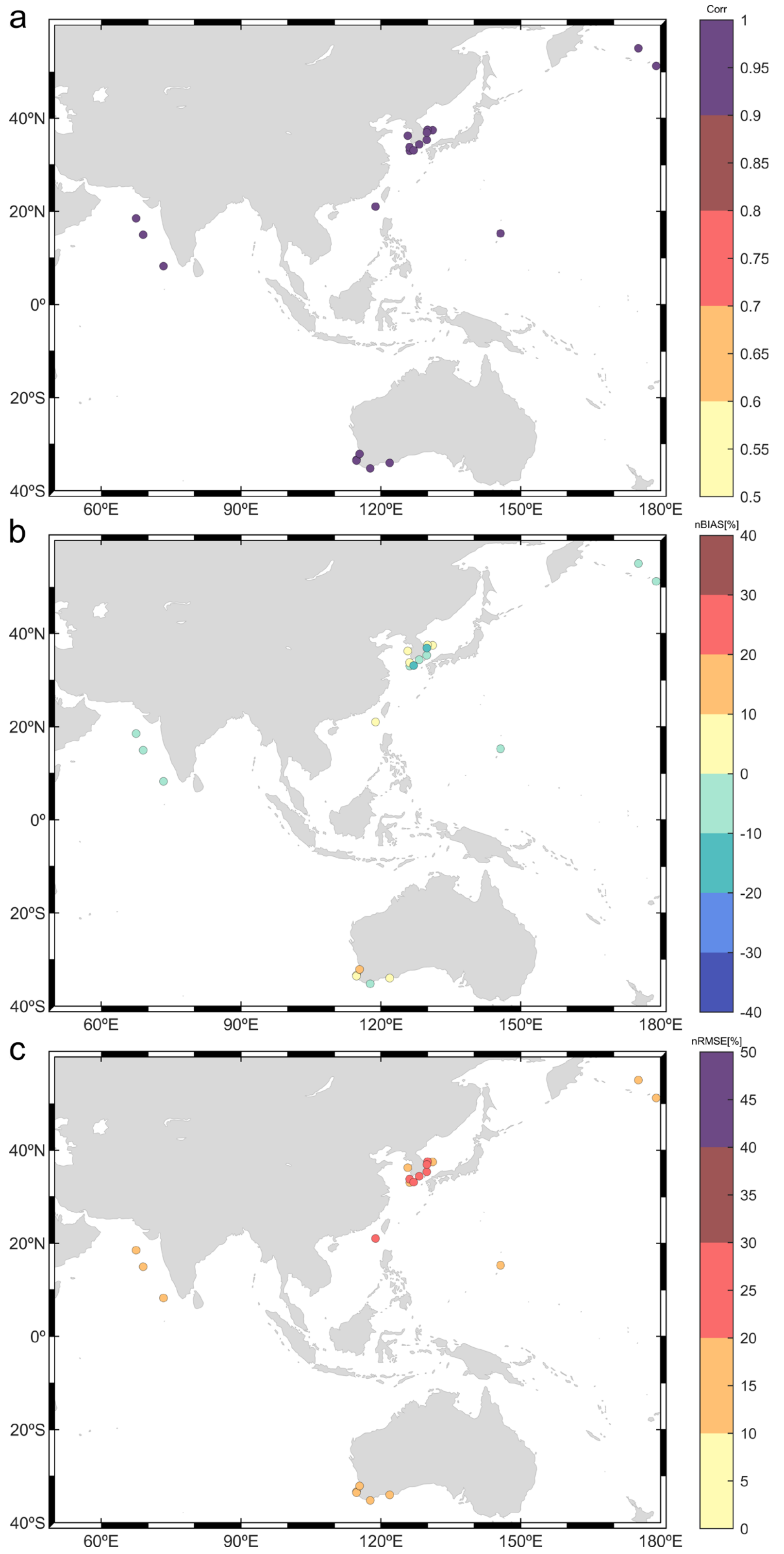

Figure SM3: *Validation of hindcast H_s against buoy data along the coasts of Asia and Australia. (a) Correlation values. (b) Normalized bias (in %) of the H_s parameter from the hindcast product relative to buoy measurements. (c) Normalized root-mean square error (in %) of the H_s parameter from the hindcast product relative to buoy measurements. The plots were generated using MATLAB R2023b (https://matlab.mathworks.com).*

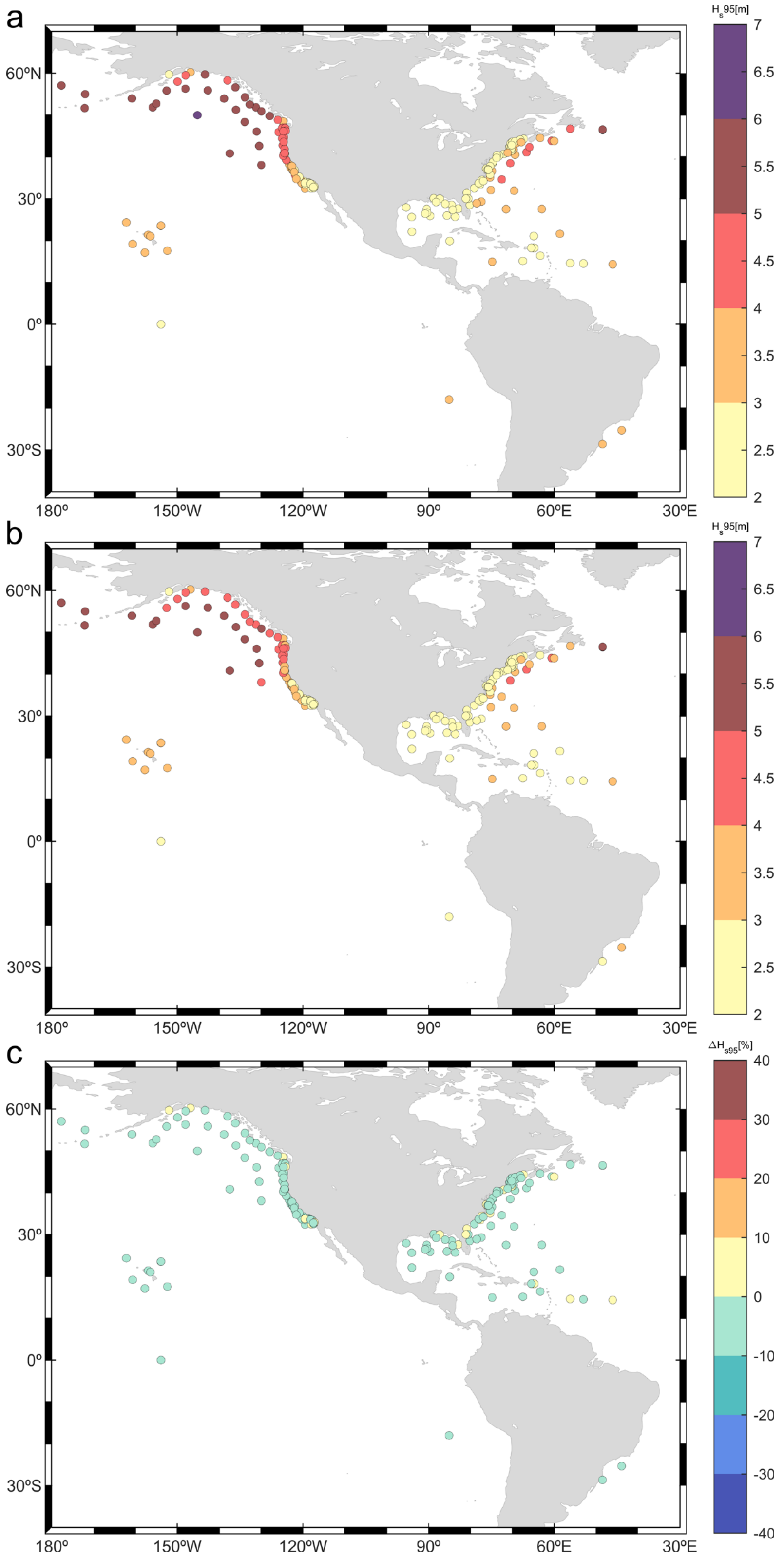

Figure SM4: *Validation of hindcast H_s95 against buoy data along the coasts of America. (a) H_s95 from buoy data (in m). (b) H_s95 from hindcast data (in m). (c) Relative difference (in %) in Hs95 between buoy and hindcast data relative to buoy measurements. The plots were generated using MATLAB R2023b (https://matlab.mathworks.com).*

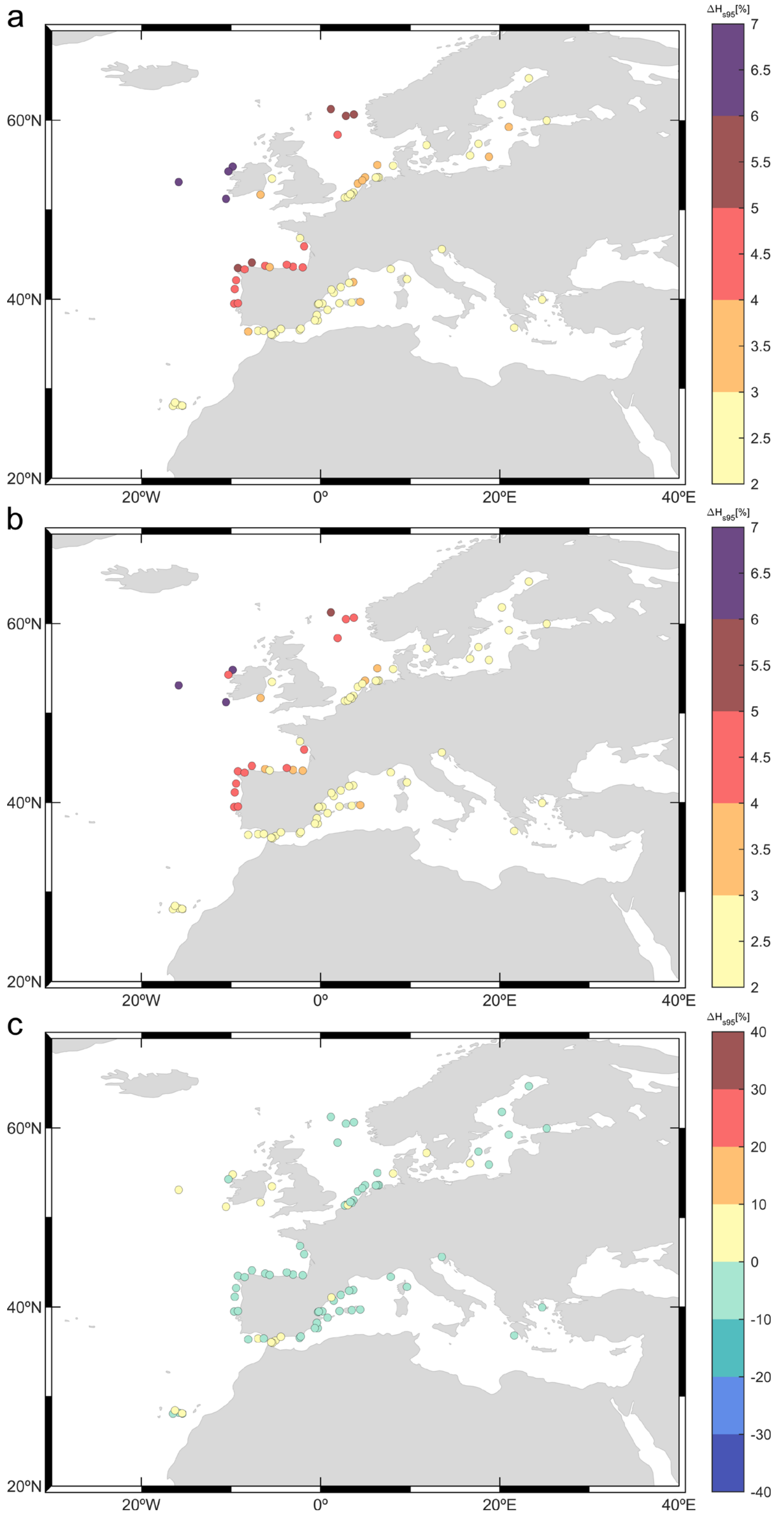

***Figure SM5**: Validation of hindcast H_s95 against buoy data along the coasts of Europe. (a) H_s95 from buoy data (in m). (b) H_s95 from hindcast data (in m). (c) Relative difference (in %) in Hs95 between buoy and hindcast data relative to buoy measurements. The plots were generated using MATLAB R2023b (https://matlab.mathworks.com).*

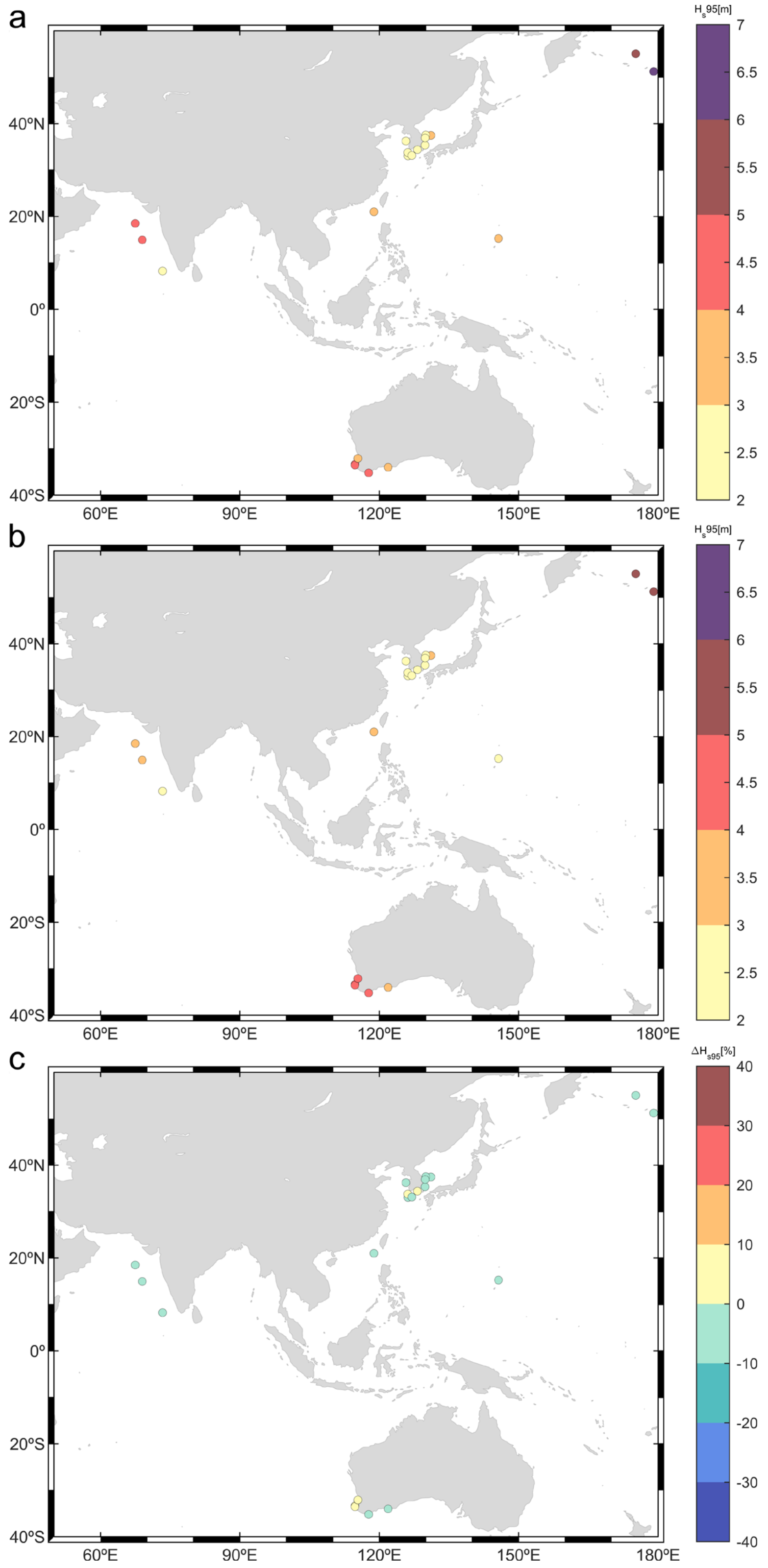

Figure SM6: *Validation of hindcast H_s95 against buoy data along the coasts of Asia and Australia. (a) H_s95 from buoy data (in m). (b) H_s95 from hindcast data (in m). (c) Relative difference (in %) in Hs95 between buoy and hindcast data relative to buoy measurements. The plots were generated using MATLAB R2023b (https://matlab.mathworks.com).*

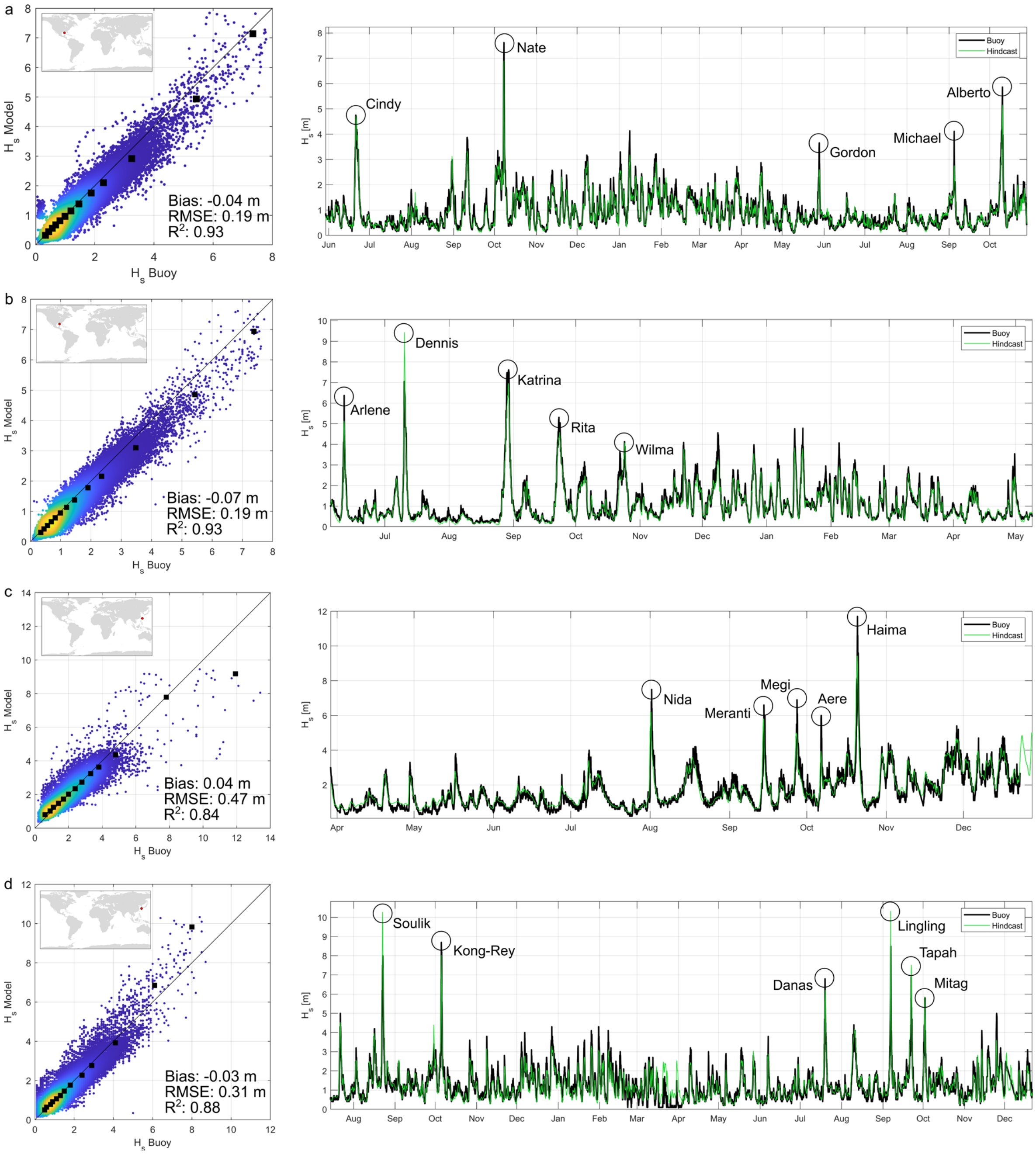

***Figure SM7**: Validation of H_s against buoy data within coastal regions affected by tropical cyclones. Scatter plots show the H_s recorded by buoys versus the H_s produced by the numerical model. Black squares show the H_s quantiles from 0.1 to 0.9, 0.95, 0.99, 0.999 and 0.9999, calculated over the available buoy data period. The plots were generated using MATLAB R2023b (https://matlab.mathworks.com).*

Additionally, a specific validation of the representation of tropical cyclones has been conducted. To that end, four buoys have been selected, two in the Gulf of Mexico and two in the south of Japan. We have validated the H_s provided by the wave model at the node closest to the position of these buoys, and qualitatively evaluated the representation of H_s peaks associated with tropical cyclones in the time series (Figure SM7).

The scatter plots show good agreement across the four buoy locations. The two buoys located in the Gulf of Mexico (upper two rows) indicate a slight underestimation of the most extreme values. It is however clear that storm events associated with tropical cyclones are present in the model. The study of the time series leads to the same conclusions. It is possible to observe how, except for a few exceptions, the most intense events, associated with tropical cyclones, show higher H_s values than the buoy records.

The buoys situated in the western Pacific and affected by swells generated by typhoons also show good agreement between both sources of information. Regarding the most extreme events, discrepancies exist between the two buoys. While one of them shows an underestimation, the other shows an overestimation. Nevertheless, the main conclusion to be drawn is, again, that the storm events generated by tropical cyclones are indeed represented in the hindcast with significant accuracy.

Coastal bathymetry

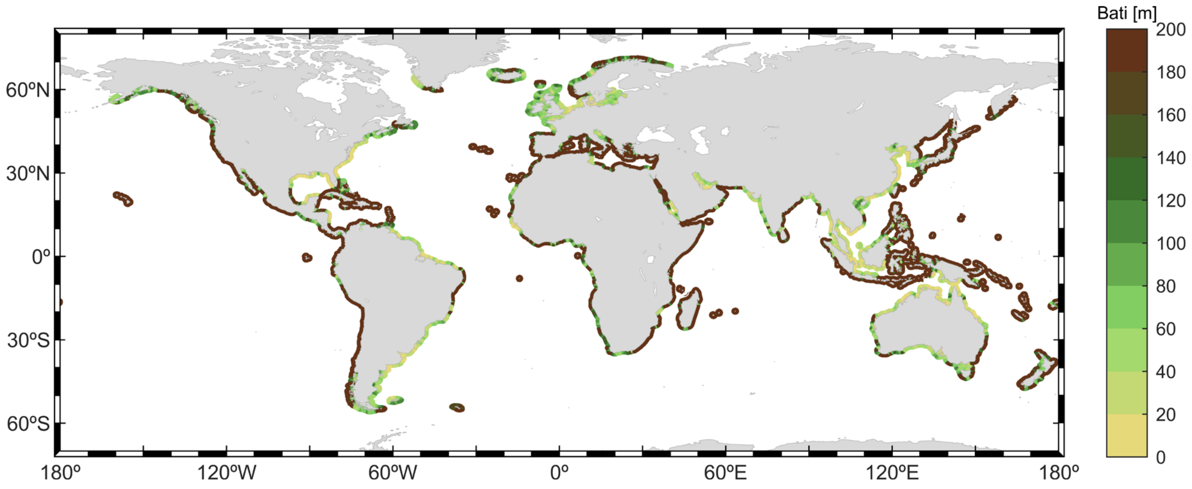

Figure SM8: *Coastal bathymetry at the location of the coastal target points*

The percentage of points in which annual maximum H_s would experience depth-induced breaking has been computed. To that end, the following wave breaking criterion has been used:

Breaking if $H_{s,b} > 0.78h_b$[1]

where $H_{s,b}$ is the breaking wave height and h_b is the breaking depth.

As a result, less than 0.1% of the coastal points meet this relationship.

Coastal mean wave climate

A brief description of the annual mean H_s wave climate (1979 to 2020) along the global coastlines is presented in Figure SM9. This will be used as a basis for further analyses and classification of the coastal wave storminess. The annual mean H_s along the global coastlines shows an overall meridional gradient (Figure SM9a). The highest values of annual mean H_s are mostly observed in

extratropical latitudes in both hemispheres. Relevant differences between eastern and western coastlines of the continents can also be seen, the latter displaying higher annual mean H_s due to the prevailing westerly winds at these latitudes and, hence, the mean eastward wave propagation[2,3]. The lowest values of annual mean H_s are found along the equatorial coastlines and in semi-enclosed basins, such as the Mediterranean and Red Seas, and in marginal seas, such as the Yellow Sea and South China Sea. The highest values of annual mean H_s are found in extratropical coastlines of the SH, a consequence of the extended fetch and the sustained generation of waves caused by the intense prevailing westerly winds[2]. In particular, the southernmost part of the Chilean coast and the east coast of Tasmania show the highest annual mean H_s values within southern extratropical coastlines, almost reaching 4 m. The coastal annual mean H_s in the extratropical latitudes of the NH show the highest values (around 3 m) in the west coasts of Ireland and Scotland. The lowest values of annual mean H_s along the global coastlines are found in the Java and Banda Seas in Indonesia, with values between 1 and 2 m, as they are sheltered from the open Indian Ocean swell waves. Figure SM9b shows the annual mean swell and wind sea predominance along the global coastlines. Around half of the global coastline shows a swell dominance with a swell to wind sea ratio exceeding 0.7, estimated as the mean proportion between m_0^s and m_0 during the whole analyzed period, which is consistent with previous studies undertaken for the deep ocean[3,4].

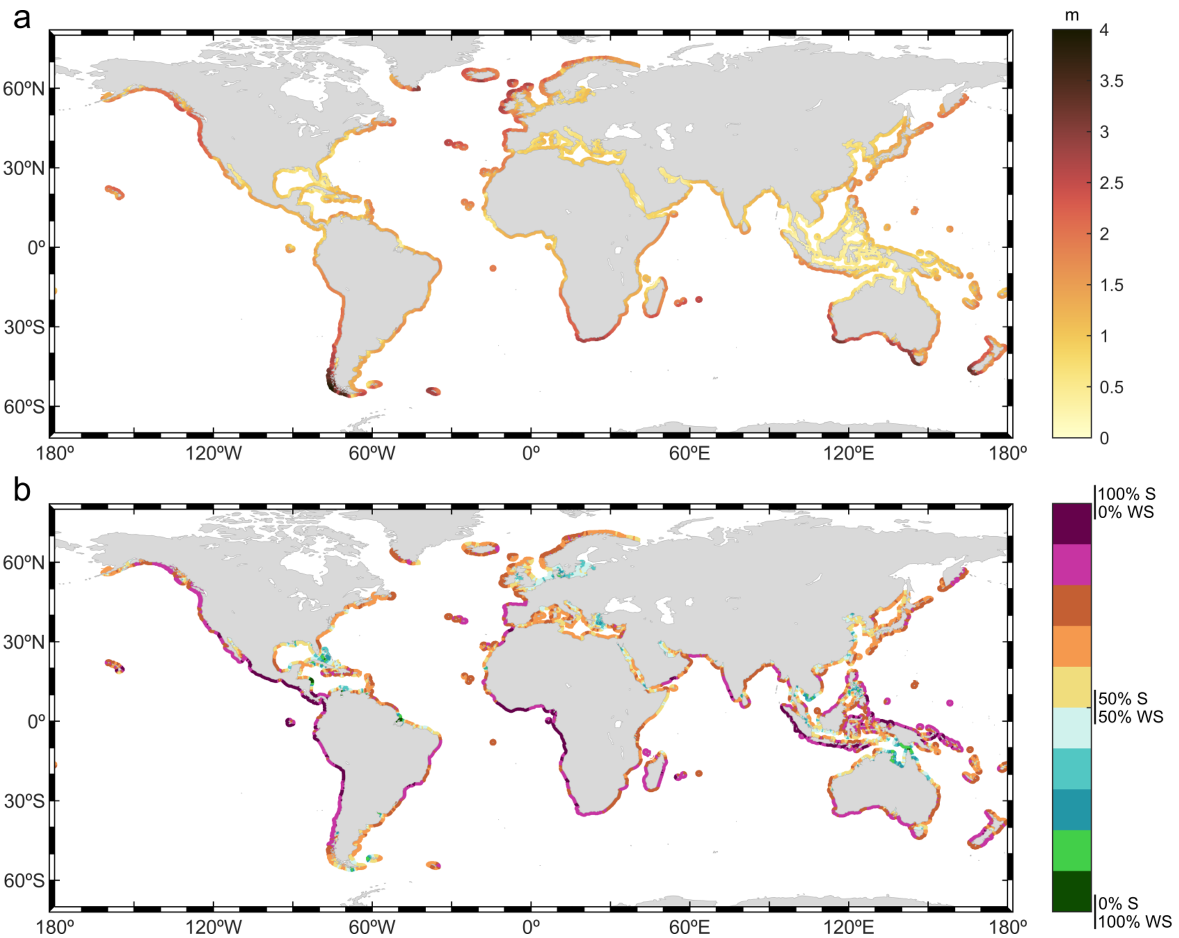

Figure SM9 *Global coastal (a) mean H_s (in m), and (b) swell (S) vs. wind sea (WS) dominance (in %) for the period 1979-2020. The plots were generated using MATLAB R2023b (https://matlab.mathworks.com).*

Assessment of wave storm characteristics

Frequency of occurrence

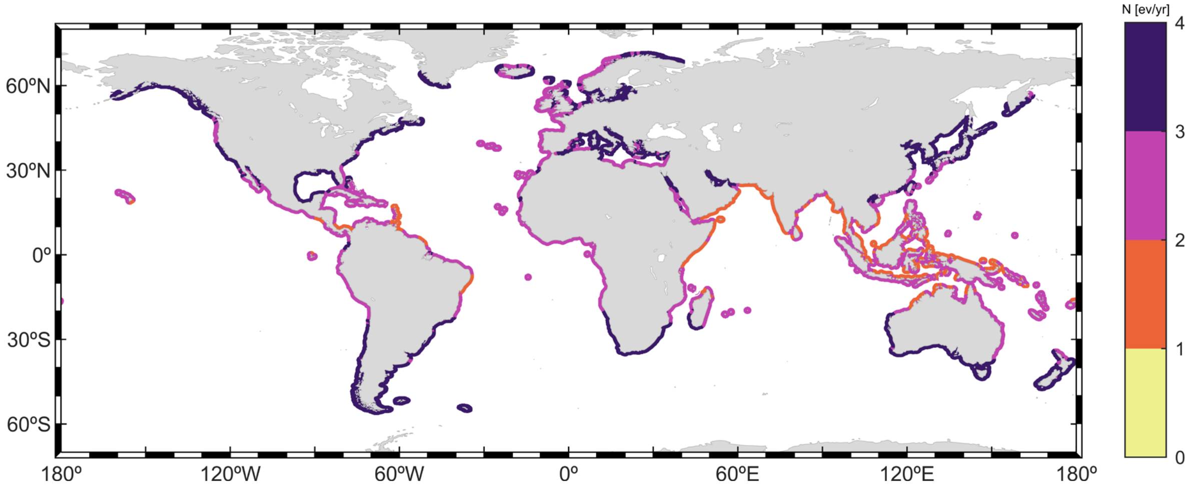

Figure SM10 *Global coastal annual mean number of severe wave storms (in events/year – ev/yr). The plots were generated using MATLAB R2023b (https://matlab.mathworks.com).*

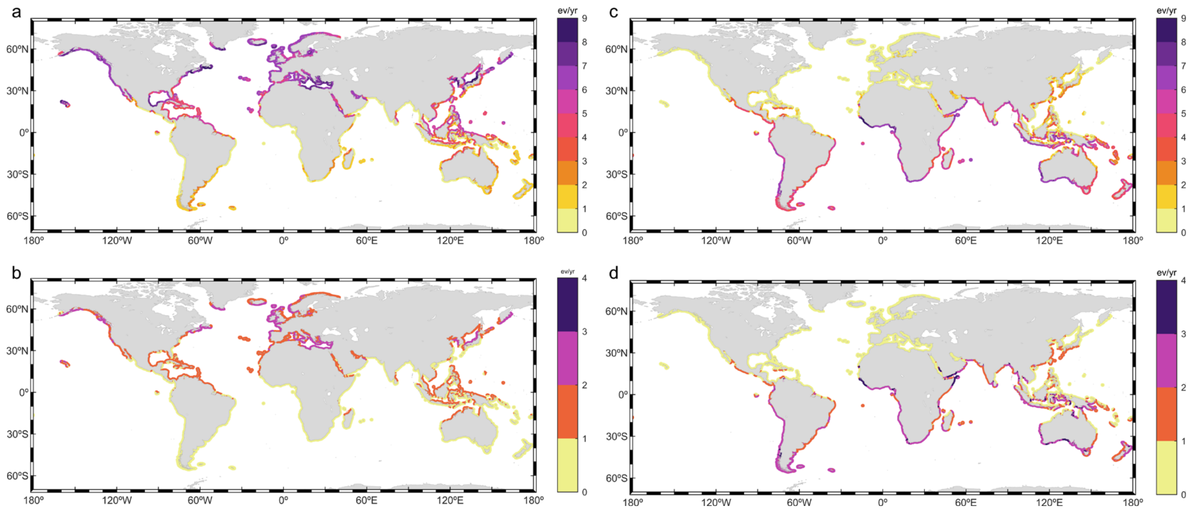

Figure SM11 *Seasonal global coastal annual mean number (in events/year - ev/yr) of (a) DJF wave storms, (b) JJA wave storms, (c) DJF severe wave storms, and (d) JJA severe wave storms. The color scales vary between the panels. The plots were generated using MATLAB R2023b (https://matlab.mathworks.com).*

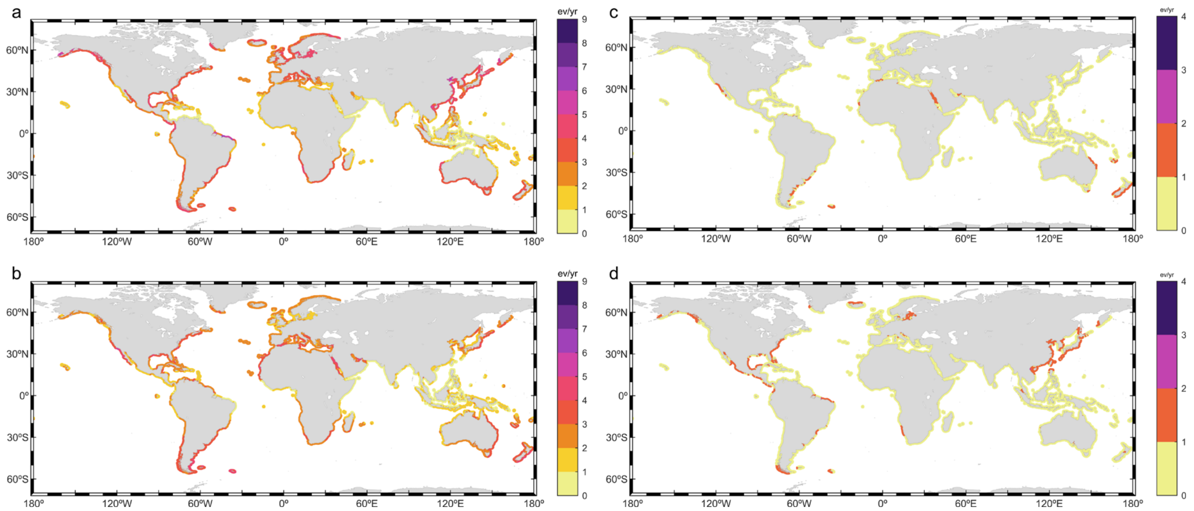

Figure SM12 *Seasonal global coastal annual mean number (events/year - ev/yr) of (a) SON wave storms, (b) MAM wave storms, (c) SON severe wave storms, and (d) MAM severe wave storms. The color scales vary between the panels. The plots were generated using MATLAB R2023b (https://matlab.mathworks.com).*

Storm duration

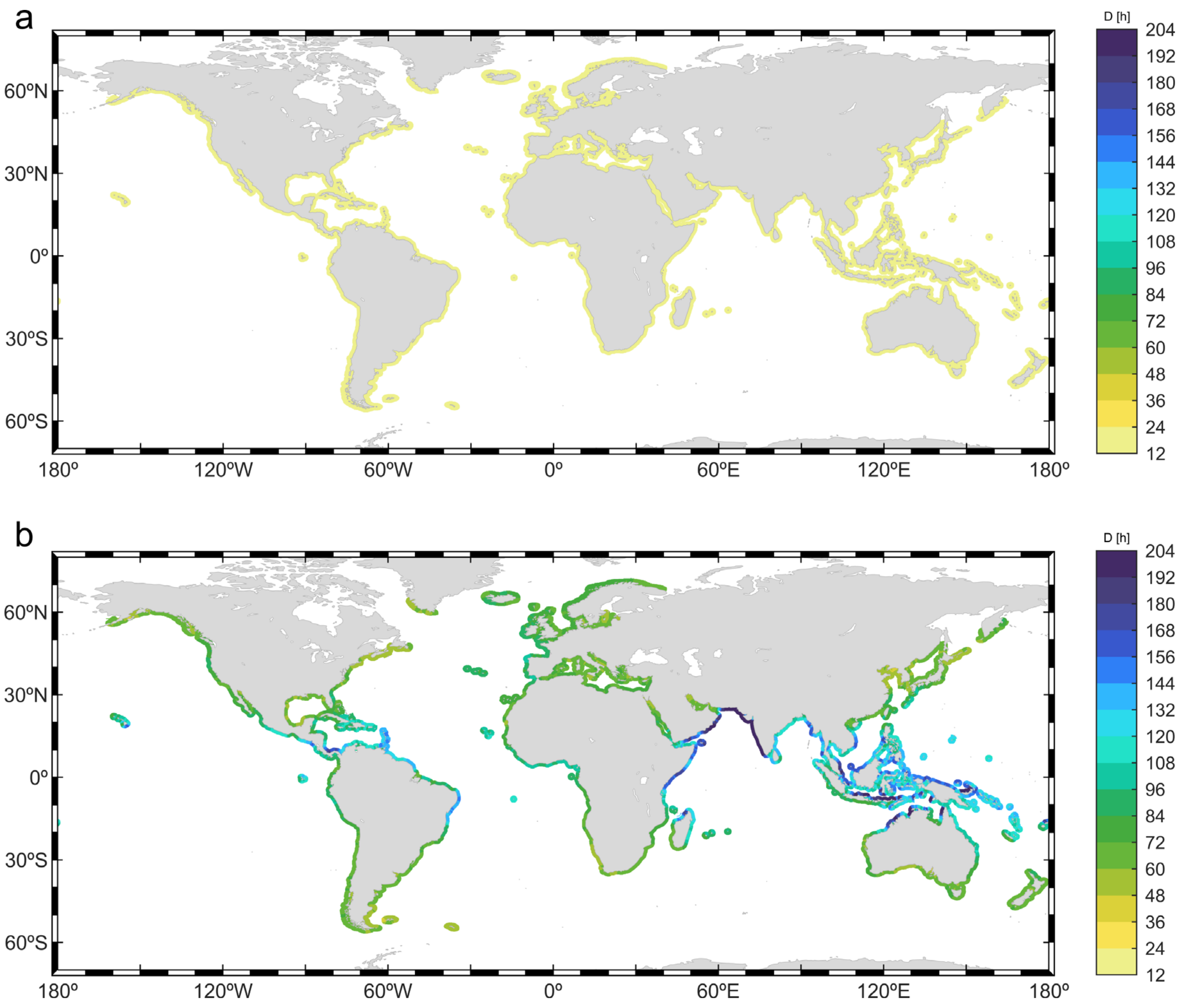

Figure SM13 *(a) 5th percentile of storm duration (in h) and (b) 95th percentile of storm duration (in hours). The plots were generated using MATLAB R2023b (https://matlab.mathworks.com).*

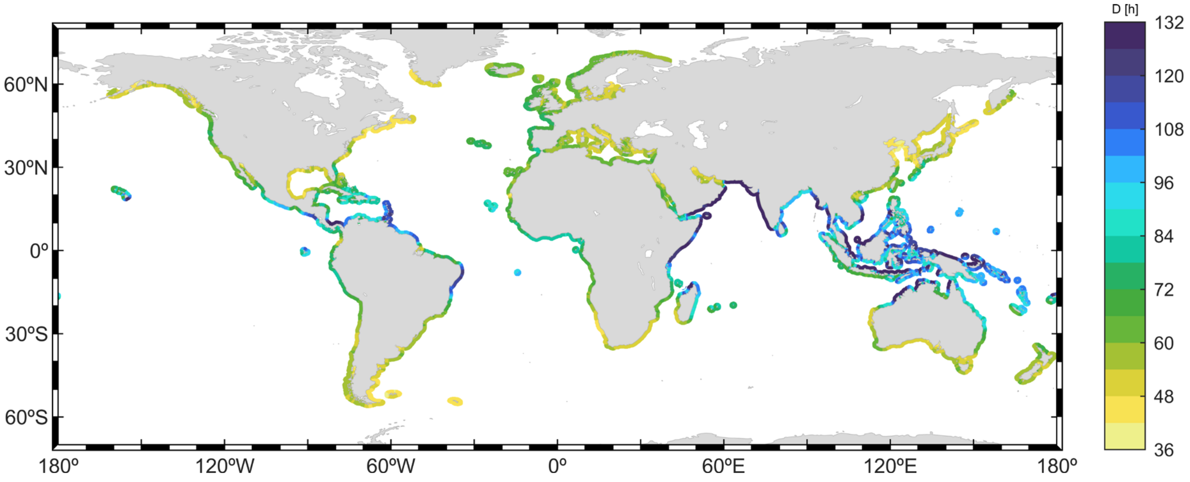

Figure SM14 *Global coastal mean duration of severe wave storms (in hours). The plots were generated using MATLAB R2023b (https://matlab.mathworks.com).*

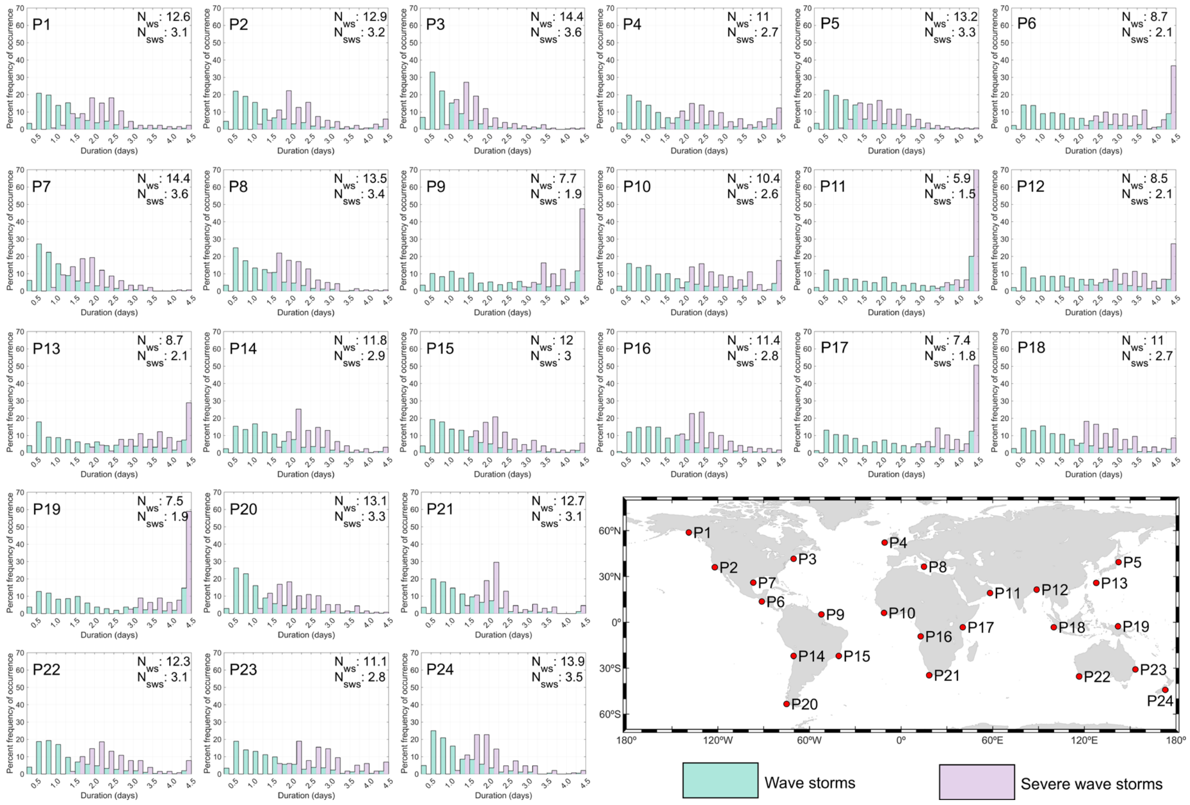

Figure SM15 *Monthly percent frequency of occurrence of wave storm durations at twenty-four key locations (P1 to P24: see map). Green bars represent wave storms and purple bars represent severe wave storms. The plots were generated using MATLAB R2023b (https://matlab.mathworks.com).*

Rank	Coastal region (shortest)	D (h)	Coastal region (longest)	D (h)
1	Yellow Sea coast	24-30	West Arabian Sea coast	>72
2	South-Africa & Namibia	24-36	Northwestern Australia	60-84
3	Northeastern USA & Eastern Canada		East Arabian Sea coast	60-76
4	Gulf of Mexico coast		East Malaysia	60-72
5	Southern - Western Australia		North Java	54-72
6	Northern Japan		Eastern Somalia	54-66
7	Northwestern America		Northern Papua-New Guinea	54-60
8	Mediterranean Europe		Myanmar	48-72
9	Southeastern America	30-36	Northern Panama	48-72
10	New Zealand		Lesser Antilles	48-60

Table SM1 *First column: Ranking order. Second and Fourth columns: coastal regions showing the top 10 shortest and longest mean durations of wave storms along the global coastlines, respectively. Third and fifth columns: frequency of occurrence (mean number of events per year – ev/yr) of wave storms.*

Storm Integrated wave parameters

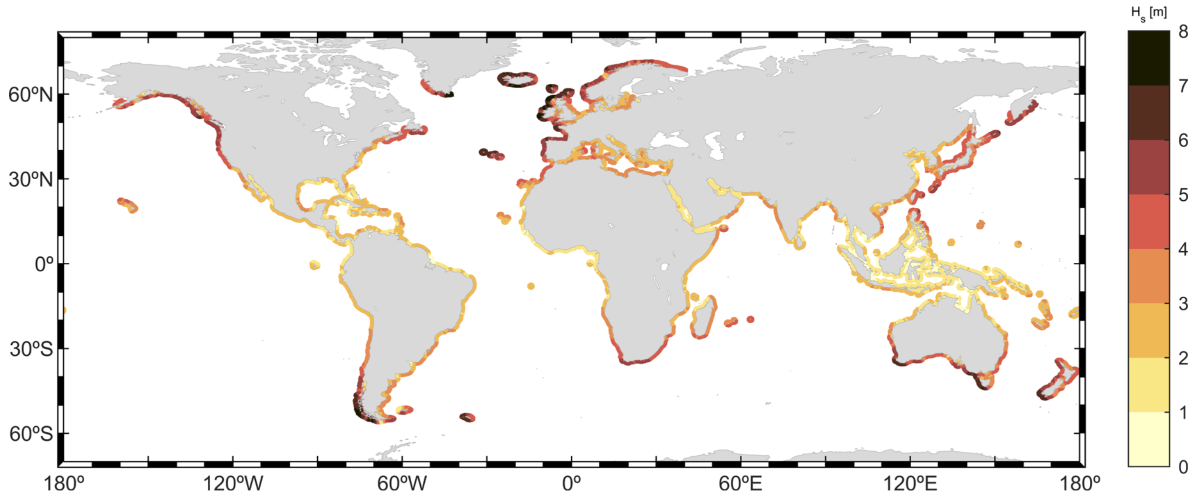

Figure SM16 *Global coastal mean H_s (in m) for severe wave storms. The plots were generated using MATLAB R2023b (https://matlab.mathworks.com).*

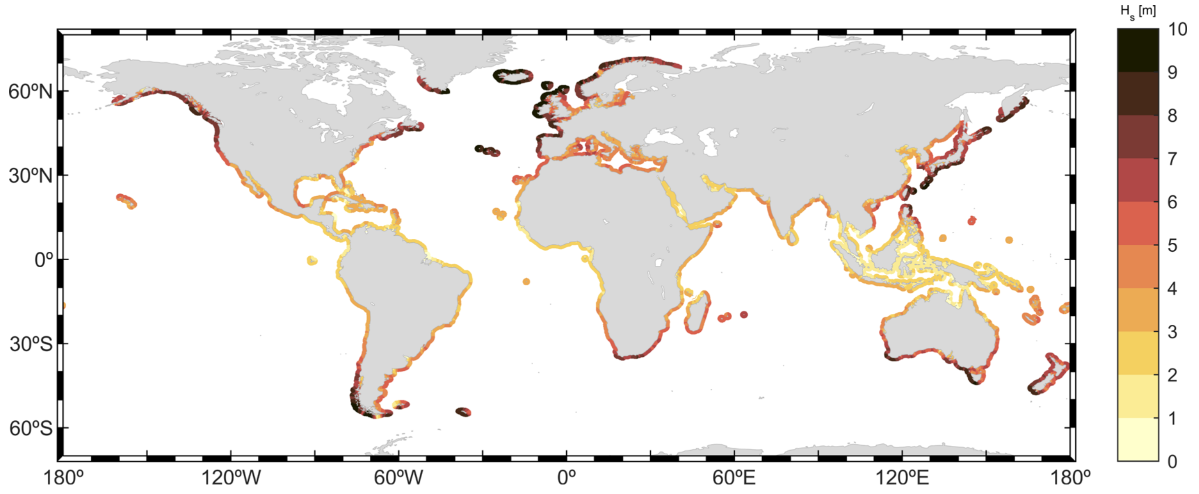

Figure SM17 *Global coastal mean annual maxima H_s (in m) registered in a wave storm. The plots were generated using MATLAB R2023b (https://matlab.mathworks.com).*

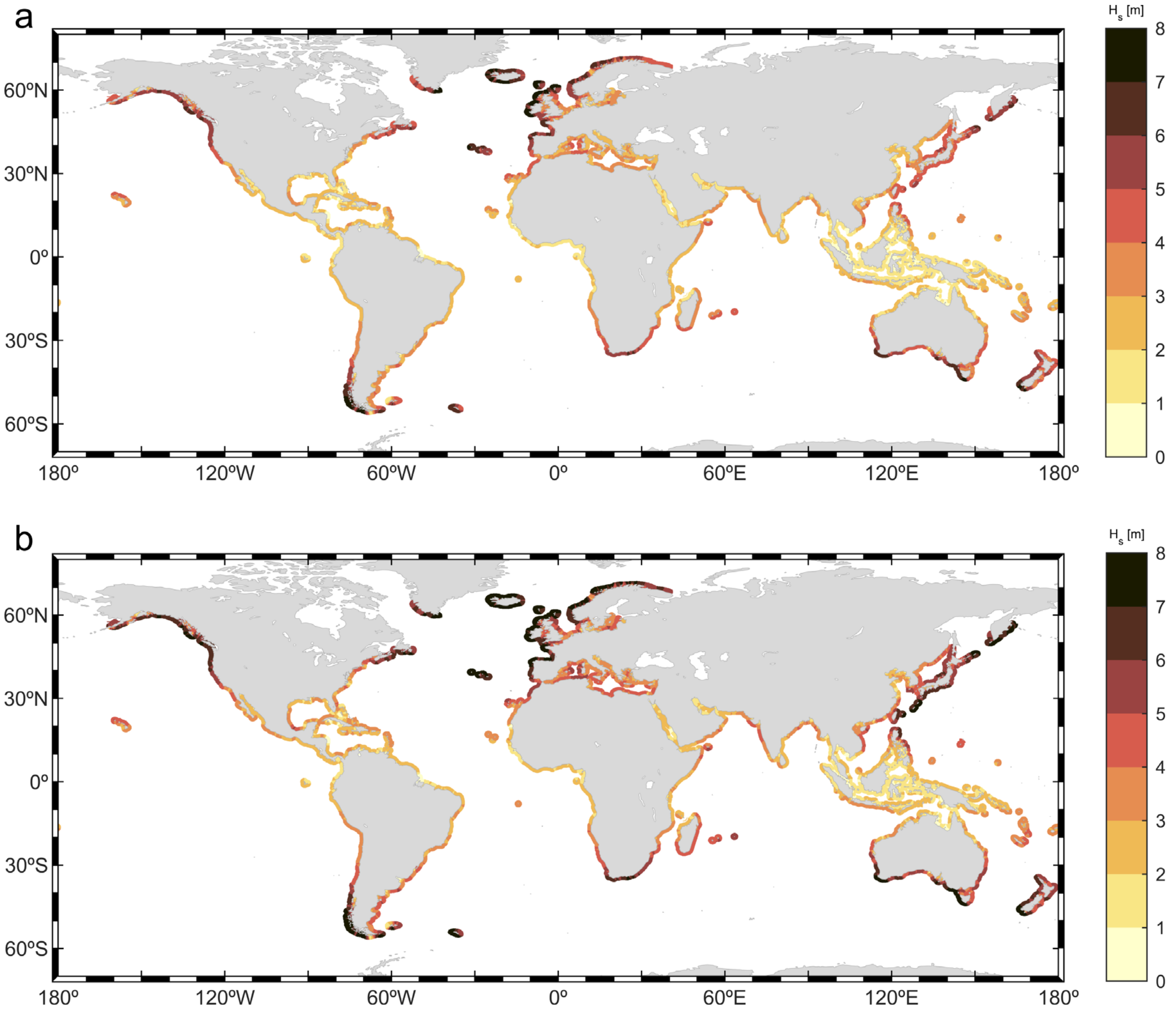

Figure SM18 *Global coastal mean storm-peak H_s (in m) for (a) wave storms and (b) severe wave storms. The plots were generated using MATLAB R2023b (https://matlab.mathworks.com).*

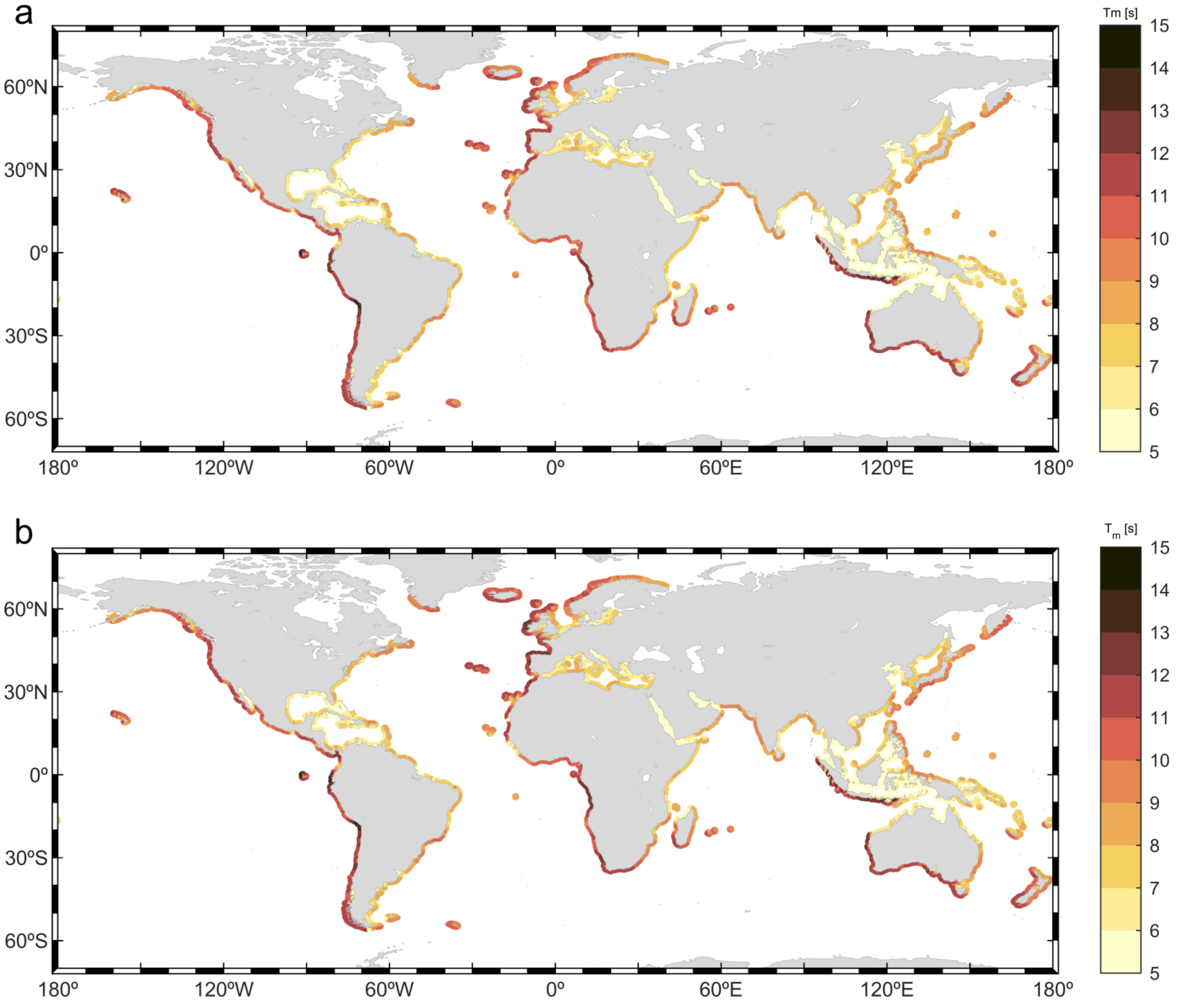

Figure SM19 *Global coastal mean T_m (in s) of (a) wave storms and (b) severe wave storms. The plots were generated using MATLAB R2023b (https://matlab.mathworks.com).*

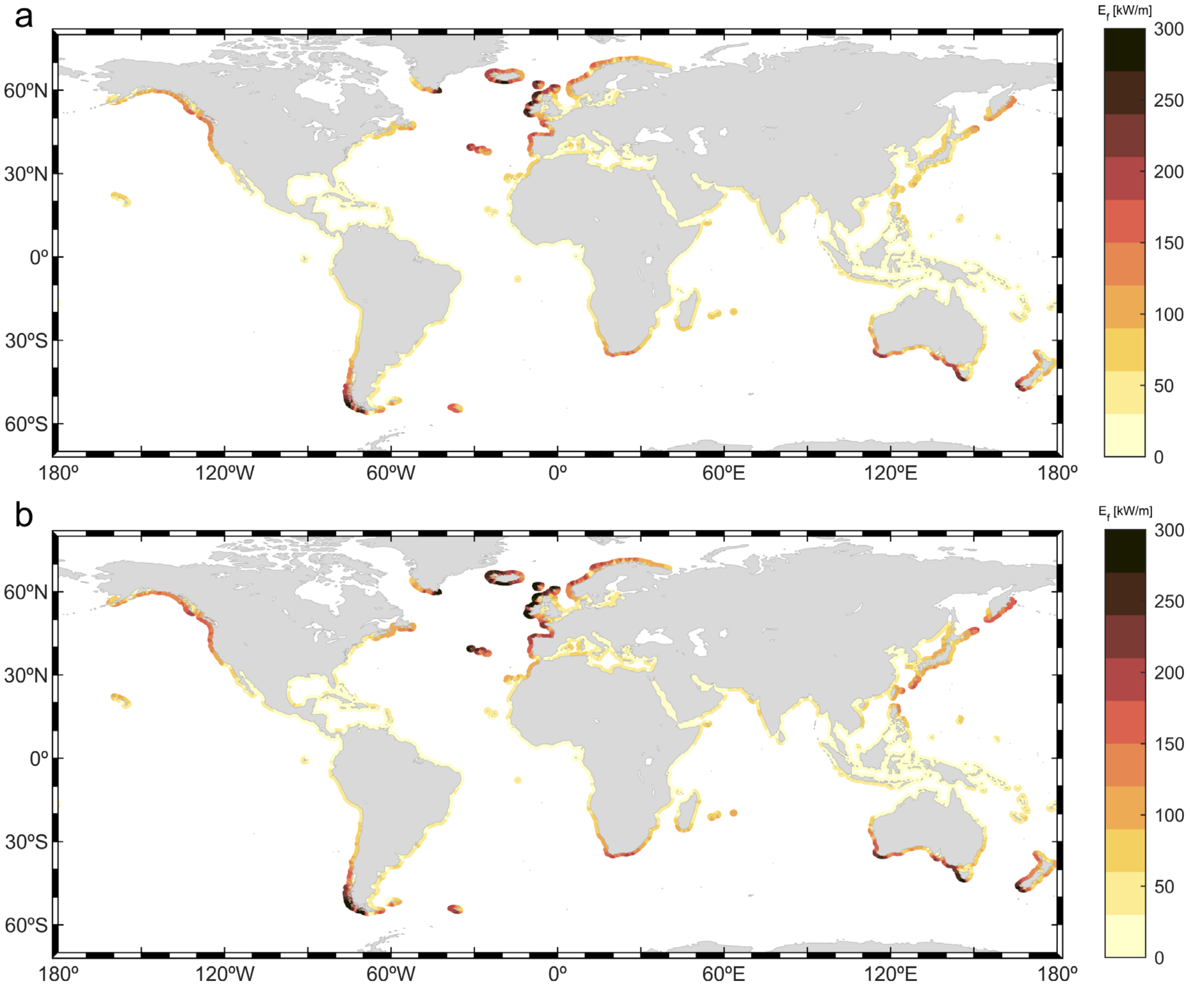

Figure SM20 *Global coastal mean E_f (in kW/m) of (a) wave storms and (b) severe wave storms. The plots were generated using MATLAB R2023b (https://matlab.mathworks.com).*

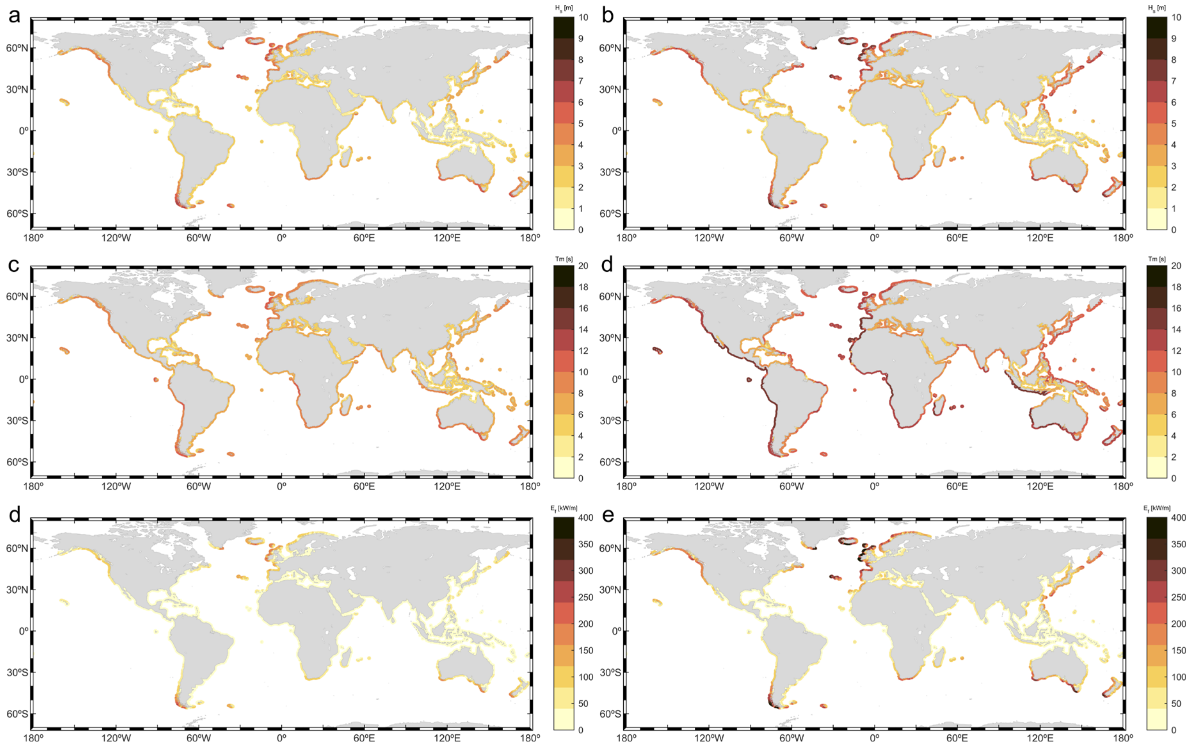

Figure SM21 *(a) 5th percentile storm H_s (in m), (b) 95th percentile storm H_s, (c) 5th percentile storm T_m, (d) 95th percentile storm T_m, (e) 5th percentile storm E_f and (f) 95th percentile storm E_f. Colorbar limits change between panels. The plots were generated using MATLAB R2023b (https://matlab.mathworks.com).*

Wind-sea vs. swell dominance

Rank	Wind-sea dominance during storms	Swell dominance during storms
1	Northwestern Europe	Western America
2	Northern Australia	Western Africa
3	Southeastern South America	Southwestern Europe
4	Gulf of Mexico / Caribbean Islands coasts	South Sumatra/Java
5	China	Northern Papua New Guinea
6	Mediterranean Sea coast	Western India
7	Red Sea coast	Eastern Australia
8	Persian Gulf coast	Southern Madagascar

Table 1 *First column: Ranking order. Second column: Top 8 coastal regions showing the clearest storm wind-sea dominance pattern. Third column: Top 8 coastal regions showing the clearest swell dominance pattern.*

Storm intensity

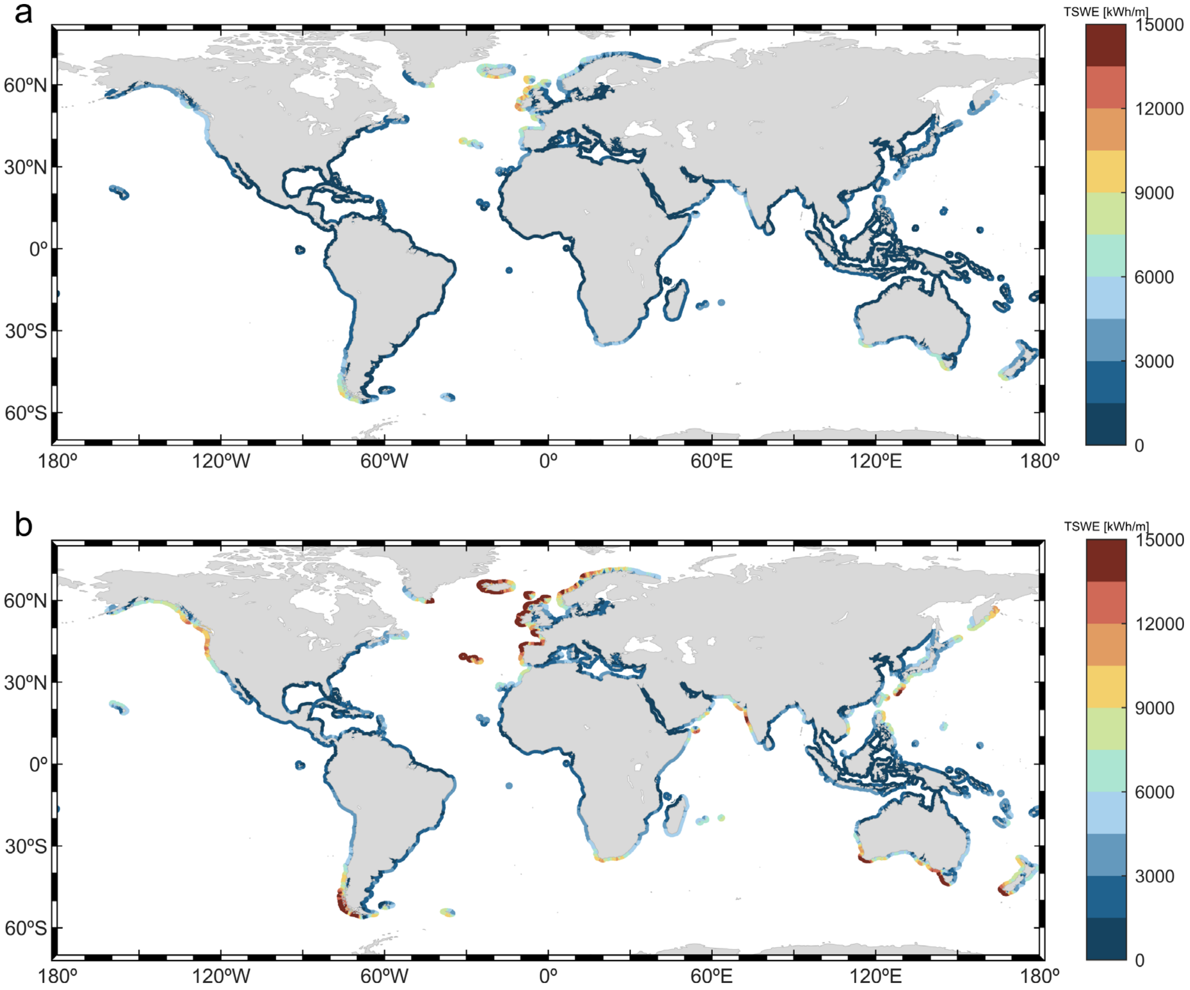

Figure SM22 *Global coastal mean total storm wave energy (TSWE, in kWh/m) of (a) wave storms and (b) severe wave storms. The plots were generated using MATLAB R2023b (https://matlab.mathworks.com).*

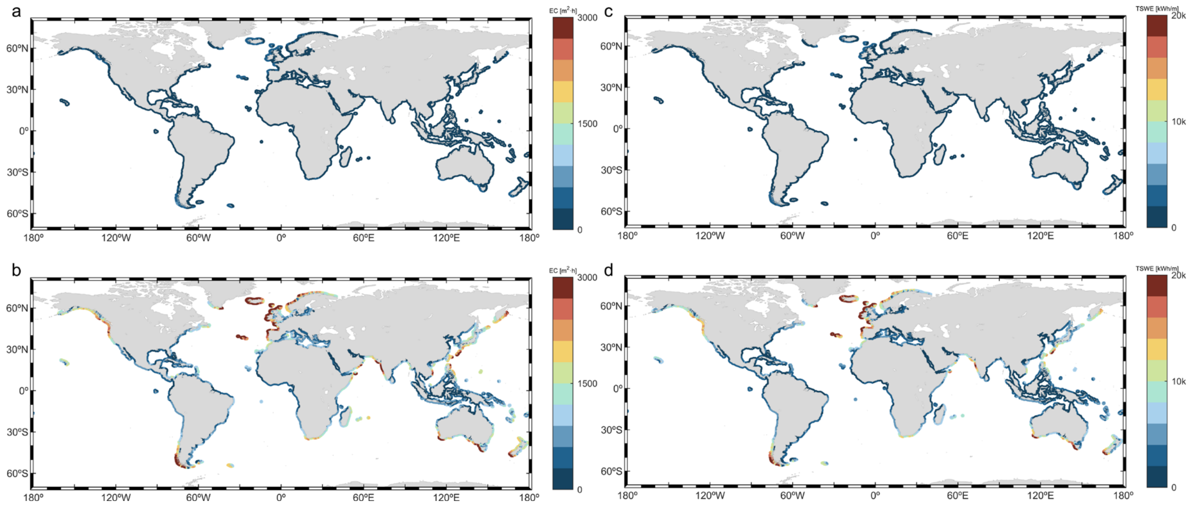

Figure SM23 *(a) 5th percentile storm energy content (EC; in m^{2}h), (b) 95th percentile storm energy content (EC; in m^{2}h), (c) 5th percentile storm total storm wave energy (TSWE; in kWh/m), (d) 95th percentile storm total storm wave energy (TSWE; in kWh/m). Colorbar limits change between panels. The plots were generated using MATLAB R2023b (https://matlab.mathworks.com).*

Storm independency

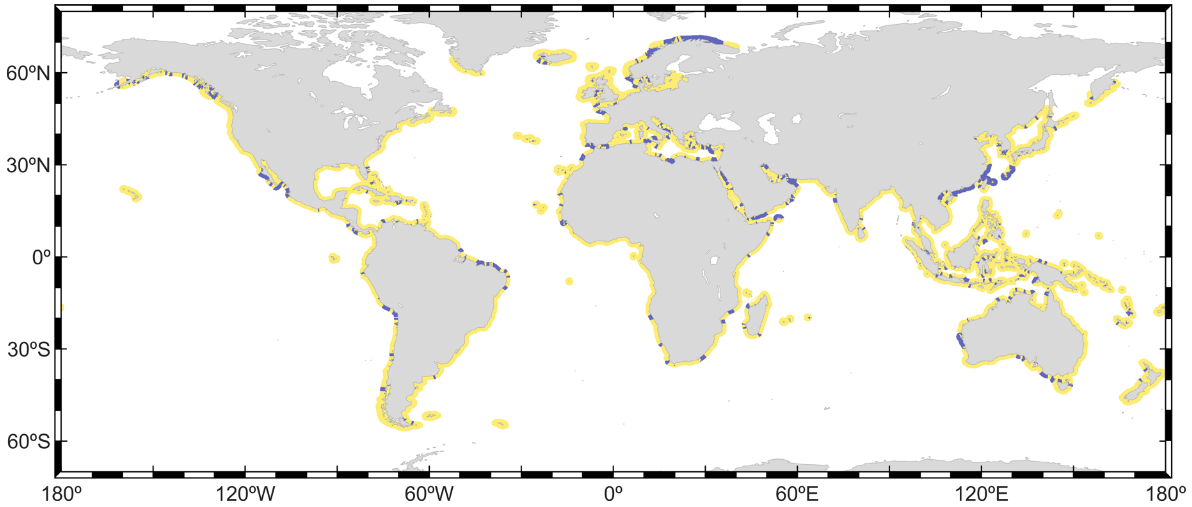

Figure SM24 *Independence analysis between consecutive storm peaks using the Kendall's Tau correlation metric. Blue indicates that the hypothesis of events being independent cannot be rejected at 5% significance level. Yellow indicates that the hypothesis of events being independent can be rejected at 5% significance level. The plots were generated using MATLAB R2023b (https://matlab.mathworks.com).*

Inter-model uncertainty

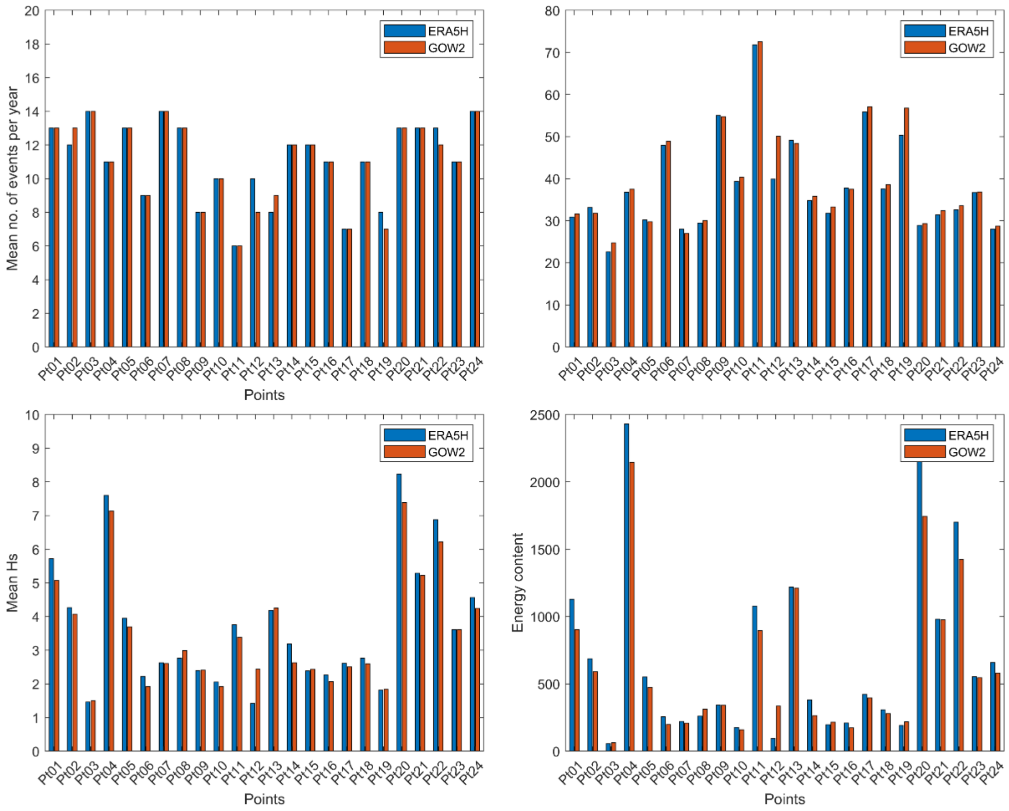

Figure SM25 *Comparison between the storm characteristics from ERA5 and GOW2 hindcasts at the 24 key points: frequency of occurrence (upper left), mean duration (upper right), mean H_s (left bottom) and energy content (right bottom). The plots were generated using MATLAB R2023b (https://matlab.mathworks.com).*

References

1. McCowan, J. XXXIX. On the highest wave of permanent type. *The London, Edinburgh, and Dublin Philosophical Magazine and Journal of Science* **38**, 351–358 (1894).

2. Young, I. R. Seasonal variability of the global ocean wind and wave climate. *International Journal of Climatology* **19**, 931–950 (1999).

3. Semedo, A., SušElj, K., Rutgersson, A. & Sterl, A. A global view on the wind sea and swell climate and variability from ERA-40. *J Clim* **24**, 1461–1479 (2011).

4. Carrasco, A., Semedo, A., Isachsen, P. E., Christensen, K. H. & Saetra, Ø. Global surface wave drift climate from ERA-40: the contributions from wind-sea and swell. *Ocean Dyn* **64**, 1815–1829 (2014).
